# Long noncoding RNA DIAPH2-AS1 promotes neural invasion of gastric cancer via stabilizing NSUN2 to enhance the m5C modification of NTN1

**DOI:** 10.1038/s41419-023-05781-5

**Published:** 2023-04-10

**Authors:** Ying Li, Yiwen Xia, Tianlu Jiang, Zetian Chen, Yikai Shen, Jie Lin, Li Xie, Chao Gu, Jialun Lv, Chen Lu, Diancai Zhang, Hao Xu, Li Yang, Zekuan Xu, Linjun Wang

**Affiliations:** 1 Division of Gastric Surgery, Department of General Surgery, The First Affiliated Hospital of Nanjing Medical University, Nanjing, 210029 Jiangsu Province China; 2 Department of General Surgery, The Affiliated Suzhou Hospital of Nanjing Medical University, Suzhou, 215008 Jiangsu Province China; 3 Jiangsu Key Lab of Cancer Biomarkers, Prevention and Treatment, Collaborative Innovation Center for Cancer Personalized Medicine, Nanjing Medical University, Nanjing, 211166 Jiangsu Province China

**Keywords:** Gastric cancer, Oncogenes

## Abstract

Neural invasion (NI) is a vital pathological characteristic of gastric cancer (GC), which correlates with tumor recurrence and a worse prognosis. Long noncoding RNAs (lncRNAs) play critical roles in various biological processes. However, the involvement of lncRNAs in NI of GC (GC-NI) remains unclear. DIAPH2-AS1 was upregulated in NI-positive GC tissues, which was confirmed by qRT-PCR. The higher expression of DIAPH2-AS1 predicted NI and worse survival for GC patients. Both in vitro and in vivo experiments, including wound-healing assay, Transwell assay, DRG-GC cells co-culture model, the mouse sciatic nerve model, and the lung metastasis model, indicated that DIAPH2-AS1 promoted the migration, invasion, and NI potential of GC cells. Mechanistically, pulldown assay and RNA immunoprecipitation assay revealed that DIAPH2-AS1 interacted with NSUN2. Subsequent experiments indicated that DIAPH2-AS1 stabilized NSUN2 from ubiquitin-proteasomal degradation via masking the K577 and K579 of NSUN2. The protection of DIAPH2-AS1 on NSUN2 improved the stability of NTN1 mRNA via m5C modification, which finally induced GC-NI. Our work uncovered DIAPH2-AS1 as a novel oncogenic lncRNA in GC-NI and validated the DIAPH2-AS1-NSUN2-NTN1 axis as a potential therapeutic target for NI-positive GC.

## Introduction

GC is the fifth most common malignant tumor and the fourth leading cause of cancer-associated death worldwide [1]. The majority of GC patients are diagnosed at the advanced stage and approximately 35.9% of GC patients are confirmed as NI by postoperative pathological diagnosis [2]. NI refers to the growth of cancer cells in a manner that surrounds nerves and implicates at least one-third of the perimeter or invades any outer membranes of the nerves including epineurium, perineurium, and endoneurium [3]. NI is associated with the worse prognosis in multiple cancers including GC [4–7]. Zhao et al. reported that NI was a feasible factor in predicting the survival outcome of GC patients [2]. Thus, elucidating the potential molecular mechanism underlying GC-NI is of great significance.

Long noncoding RNAs (lncRNAs) are transcripts that are longer than 200 nucleotides with limited or no potential for coding proteins [8]. LncRNAs were initially considered useless products generated during the process of transcription. However, in the past decades, accumulating studies demonstrated their vital functions in the biological processes of various cancers [9–11]. Although the past decades have witnessed massive progress in revealing the function of lncRNAs in GC [12], the participation of lncRNAs in the process of GC-NI remains unexplored.

Axon guidance molecules were initially regarded as guiding and promoting the development of the nerve system, such as inducing synapse formation [13]. Moreover, increasing studies suggested that these axon guidance molecules might serve as essential factors in diverse tumors [14, 15]. As an important axon guidance factor, NTN1 is also reported to play a fundamental role in accelerating GC aside from its functions in the nervous system [16–18]. Moreover, our previous research also demonstrated that NTN1 facilitates the process of GC-NI [19]. Nevertheless, the specific regulatory mechanisms of NTN1 in GC remain to be further explored.

Herein, we explored the lncRNA profile in GC-NI, which identified the upregulation of DIAPH2-AS1 in NI-positive GC tissues. DIAPH2-AS1 interacts with NSUN2 and stabilizes NSUN2 from ubiquitin-proteasome pathway-mediated degradation. The protection of DIAPH2-AS1 on NSUN2 improves the stability of NTN1 mRNA via the m5C modification, which finally induces GC-NI. Our study provides a new diagnostic biomarker for GC-NI and novel insights into the molecular mechanism of GC-NI.

## Results

### DIAPH2-AS1 is upregulated in NI-positive GC patients and correlated with GC-NI and poor prognosis of GC

**Fig. 1:**
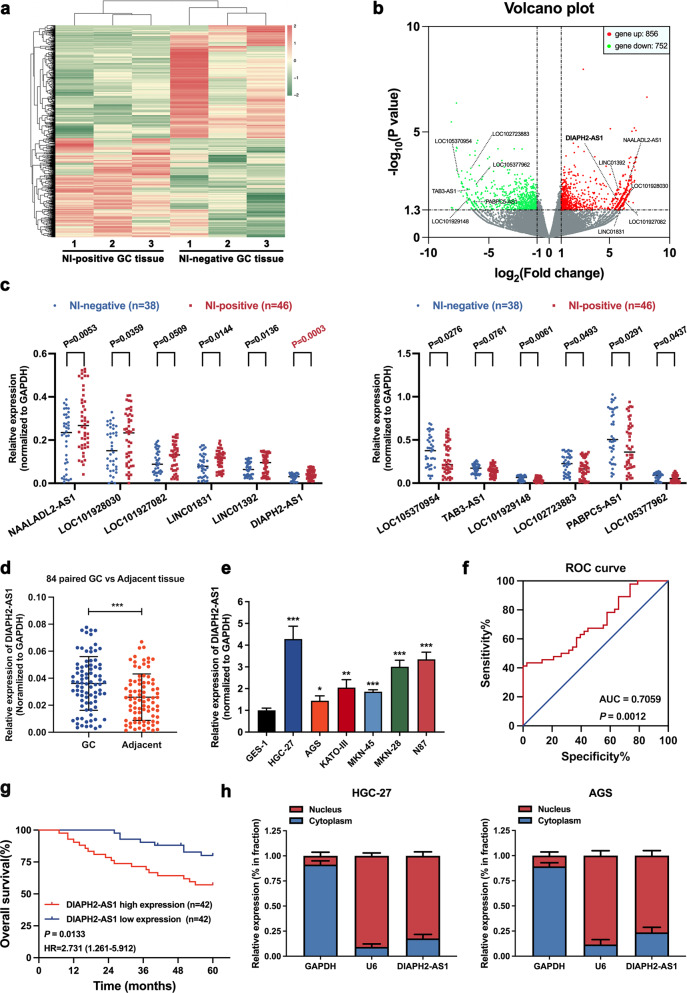
DIAPH2-AS1 is upregulated in NI-positive GC patients and correlated with GC-NI and poor prognosis of GC. **a** Heatmap of high throughput sequencing analysis showing DEGs. **b** Volcano plot of DEGs. The top six upregulated (red dots) or downregulated (green dots) lncRNAs were marked. **c** Expression of the top six upregulated or downregulated lncRNAs in enlarged 46 NI-positive tissue samples versus 38 NI-negative GC tissue samples detected by qRT-PCR. **d** DIAPH2-AS1 level of 84 pairs GC and adjacent normal tissues quantified by qRT-PCR. **e** DIAPH2-AS1 expression level of GES-1 and six GC cell lines including HGC-27, AGS, KATO-III, MKN-45, MKN-28, and NCI-N87 quantified by qRT-PCR. **f** ROC curve was drawn according to the DIAPH2-AS1 expression of 84 GC patients. **g** Kaplan–Meier survival analysis of OS of 84 GC patients by log-rank (Mantel-Cox) test. **h** Nuclear-cytoplasmic fraction assay was utilized to quantify the distribution of DIAPH2-AS1 in the cytoplasm and nucleus in HGC-27 and AGS cells. GAPDH and U6 were set as the positive control in the cytoplasm and nucleus, respectively. Data and error bars were shown as mean ± SD of triplicate independent replicate experiments and all data were analyzed by Student’s t test (*P < 0.05; **P < 0.01; ***P < 0.001).

Transcriptome sequencing was performed using 3 NI-positive and 3 NI-negative GC samples to unveil the potential differentially expressed genes (DEGs) that participated in GC-NI (Sequencing details are found in Supplementary Table S4). A batch of differentially expressed DEGs (*P* value < 0.05, log_2_FC > 2) was detected (Fig. 1a, b). We mainly focused on lncRNAs as their roles in GC-NI is still unclear. The top six upregulated or downregulated lncRNAs showed in Fig. 1b were further verified via qRT-PCR in an expanded sample cohort of 46 NI-positive versus 38 NI-negative GC tissues. Among the 12 candidate lncRNAs, upregulation of DIAPH2-AS1 in NI-positive GC tissues compared with NI-negative GC tissues showed the most significant statistical difference (Fig. 1c). Consequently, DIAPH2-AS1 was chosen for further exploration. Moreover, by qRT-PCR, we found that DIAPH2-AS1 was also remarkably upregulated in 84 GC tissues compared with the paired adjacent tissues (Fig. 1d). In addition, the expression of DIAPH2-AS1 was also quantified in several GC cell lines, including HGC-27, AGS, KATO-III, MKN-45, MKN-28, and NCI-N87. Compared with GES-1 (the normal human gastric epithelial cells), DIAPH2-AS1 was universally upregulated in the GC cell lines as mentioned above (Fig. 1e). Besides, the result of qRT-PCR revealed the highest DIAPH2-AS1 abundance in HGC-27 and the lowest in AGS. Therefore, we selected HGC27 and AGS for subsequent experiments.

Next, to elucidate the clinical significance of DIAPH2-AS1 in GC, we analyzed the correlation between the expression level of DIAPH2-AS1 and the clinicopathologic parameters of 84 GC patients. As shown in Table 1, higher expression of DIAPH2-AS1 was significantly correlated with the worse T stage, the more advanced tumor stage, and the higher incidence of GC-NI. In addition, the receiver operation characteristic (ROC) curve showed that DIAPH2-AS1 might serve as a diagnostic biomarker for GC-NI (Fig. 1f). More importantly, the Kaplan–Meier survival curve indicated that the higher level of DIAPH2-AS1 was closely correlated with the worse OS of GC patients (Fig. 1g). Above all, DIAPH2-AS1 may be a feasible biomarker in diagnosing GC-NI and predicting the prognosis of GC patients.

**Table 1:** Correlation between DIAPH2-AS1 expression and clinicopathologic parameters of 84 GC patients.

Characteristic	Number of cases (*n* = 84)	Number of cases		*P* value
		DIAPH2-AS1^low^ (*n* = 42)	DIAPH2-AS1^high^ (*n* = 42)	
*Age (years)*				
<60	20	10	10	>0.9999
≥60	64	32	32	
*Gender*				
Male	63	33	30	0.615
Female	21	9	12	
*Tumor size (cm)*				
<3	21	10	11	>0.9999
≥3	63	32	31	
*Tumor site*				
Proximal	36	17	19	0.826
Nonproximal	48	25	23	
*T stage*				
Tis-T2	21	15	6	0.042*
T3-T4	63	27	36	
*Lymph node status*				
Negative (N0)	37	22	15	0.187
Positive (N1–N3)	47	20	27	
*Neural invasion*				
Negative	38	26	12	0.004**
Positive	46	16	30	
*Tumor differentiation*				
Well-moderate	31	20	11	0.070
Poor-signet	53	22	31	
*Tumor stage*				
I–II	33	23	10	0.007**
III	51	19	32	

*P* < 0.05 was considered significant (*X*^*2*^ test). *P < 0.05; **P < 0.01.

Besides, to rule out the coding potential of DIAPH2-AS1, we used the online tools Coding Potential Calculator (CPC) (http://cpc.cbi.pku.edu.cn/) and RNA Coding Potential Assessment Tool (CPAT) (http://lilab.research.bcm.edu/cpat/index.php) [20, 21]. Both results showed that DIAPH2-AS1 possessed no potential for coding proteins (Supplementary Fig. S1b, c). A great number of studies reported that the subcellular location plays a decisive function in the biological effects of lncRNAs. Subcellular fraction assay demonstrated that DIAPH2-AS1 was mainly amplified via the fraction of the nucleus component of HGC-27 and AGS cells (Fig. 1h), which indicated that DIAPH2-AS1 was mainly located in the nucleus.

### DIAPH2-AS1 promotes the migration, invasion, and NI potential of GC cells in vitro

**Fig. 2:**
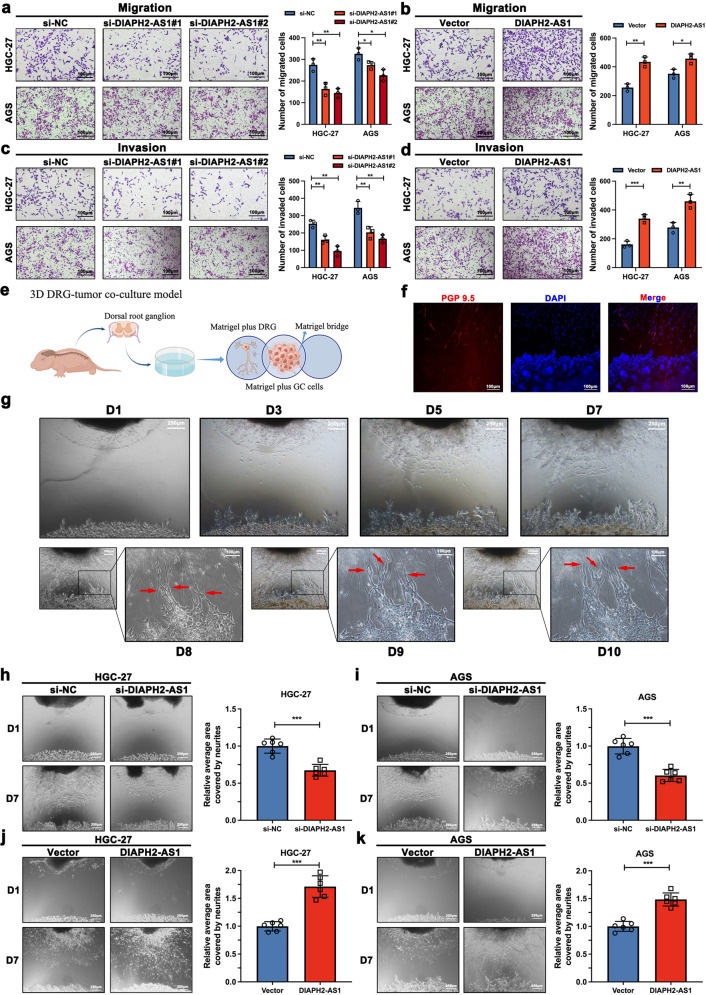
DIAPH2-AS1 promotes the migration, invasion, and NI potential of GC cells in vitro. **a**–**d** Transwell assay were performed to assess the migration and invasion ability of HGC-27 and AGS cells which were transfected with indicated siRNAs or plasmids. Representative images and corresponding statistical histograms were shown. scale bar: 100μm. Data and error bars were shown as mean ± SD of triplicate independent replicate experiments and all data were analyzed by Student’s t test. **e** Simplified schematic graph of the DRG-GC cells co-culture model was drawn by Figdraw. **f** Immunofluorescence using anti-PGP 9.5 was performed, red (PGP 9.5) represents neurites of DRGs and blue (DAPI) represents the nucleus of neurites and GC cells; scale bar: 100 μm. **g** Continuously photographing of day1, day3, day5, day7, day8, day9, and day10 of DRG-GC cells co-culture model. scale bar: 250 μm or 100 μm. **h**–**k** DRG-GC cells co-culture model was utilized to assess the NI potential of GC cells. HGC-27 and AGS cells that were transfected with si-NC, si-DIAPH2-AS, empty vector, or DIAPH2-AS1 were used. Representative D1 (day1) and D7 (day7) images and corresponding statistical diagrams were exhibited. NI potential was measured according to the relative area covered by neurites calculated by ImageJ. Scale bar: 250 μm. Data were shown as mean ± SD of six independent replicate experiments and all data were analyzed by Student’s t test (*P < 0.05; **P < 0.01; ***P < 0.001).

To explore whether DIAPH2-AS1 altered the progression of GC-NI, firstly, we constructed three small interfering RNAs (siRNA) targeting DIAPH2-AS1 and the DIAPH2-AS1-overexpression plasmid. The efficacies of siRNAs and DIAPH2-AS1-overexpression plasmid were verified via qRT-PCR (Supplementary Fig. S2a, b). Among three siRNAs, we selected si-DIAPH2-AS1#1 and si-DIAPH2-AS1#2, which showed the more substantial effects of knockdown in HGC-27 and AGS cells. Then, gain or loss-of-function experiments were conducted. The wound-healing assay showed that transfection of si-DIAPH2-AS1#1 or si-DIAPH2-AS1#2 impaired the migration capability of HGC-27 and AGS cells. Conversely, overexpression of DIAPH2-AS1 significantly strengthened the healing speed of the scratch (Supplementary Fig. S2c). Next, the Transwell assay suggested that knockdown of DIAPH2-AS1 remarkably decreased the number of migrated and invaded HGC-27 and AGS cells (Fig. 2a, c). Compared with the empty vector, DIAPH2-AS1 overexpression increased the migration and invasion abilities of HGC-27 and AGS cells (Fig. 2b, d). Through these experiments, we concluded that DIAPH2-AS1 promoted the migratory and invasive ability of GC cells.

Next, to explore whether DIAPH2-AS1 participated in the process of GC-NI, as the diagrammatic sketch created by Figdraw showed (Fig. 2e), we established a refined DRG-GC cells co-culture model as described in the previous works [22, 23]. Meanwhile, immunofluorescence using the specific antibody against PGP9.5 validated the successful dissection of DRGs (Fig. 2f). Consecutive photographing revealed that GC cells in the co-culture model were intended to be attracted by neurites and grew in a way along the neurites upon contact with each other (red arrows) (Fig. 2g). As the DRG-GC cells co-culture model showed (Fig. 2h–k), overexpression of DIAPH2-AS1 increased the area that neurites covered in the co-culture model and silence of DIAPH2-AS1 decreased the area.

Overall, we confirmed that DIAPH2-AS1 promoted the migratory, invasive, and NI potential of GC cells in vitro.

### DIAPH2-AS1 promotes metastatic biological behavior and NI potential of GC cells in vivo

**Fig. 3:**
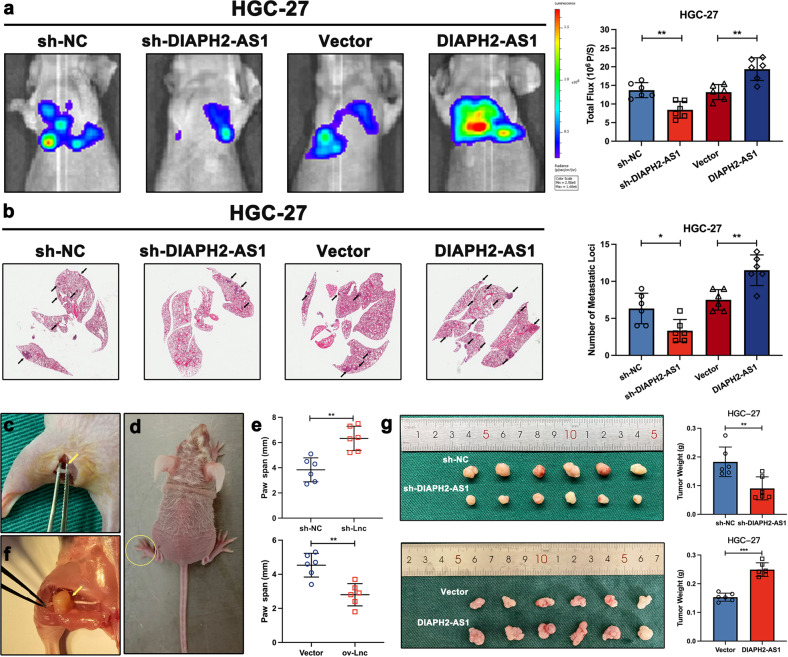
DIAPH2-AS1 promotes metastatic biological behavior and NI potential of GC cells in vivo. **a** Representative image of bioluminescent images and the statistical charts were shown (*n* = 6). **b** H&E staining of lung tissues and quantification of the number of metastatic lung foci were shown. **c** The representative image showed the surgically exposed sciatic nerves. **d** Four weeks later, the hindlimb function was damaged. **e** The statistical graphs exhibiting paw spam of mice of the mouse sciatic nerve model were displayed. **f** After four weeks, the xenograft showed a tendency to grow along the sciatic nerve. **g** Xenograft neoplasms of the mouse sciatic nerve model and quantification of tumor weight were shown. Data and error bars were shown as mean ± SD of six independent replicate experiments and all data were analyzed by Student’s t test (*P < 0.05; **P < 0.01; ***P < 0.001).

To clarify the potential biological function of DIAPH2-AS1 in vivo, we first established a metastatic lung colonization model using BALB/c nude mice by inoculating HGC-27 cells that were stably transfected with the indicated lentivirus or plasmids co-transfected with a luciferase plasmid through the tail vein. The IVIS Spectrum Imaging System was utilized to assess the lung lesion, and then these mice were sacrificed to harvest and stain the lung tissues by H&E staining five weeks later. The results showed that DIAPH2-AS1 knockdown weakened lung metastasis, while DIAPH2-AS1 overexpression exhibited the opposite effects (Fig. 3a). H&E staining also confirmed that the number of metastatic nodules was increased by transfection of DIAPH2-AS1 but decreased by silencing DIAPH2-AS1 (Fig. 3b).

In addition, as shown in Fig. 3c, by injecting HGC-27 cells into the periphery of the sciatic nerve, we constructed a mouse sciatic nerve model to evaluate the function of DIAPH2-AS1 on GC-NI in vivo. The hindlimb behavior and the tumor weight both served as evaluation standards of the NI severity. Four weeks later, the hindlimb function was damaged (Fig. 3d, e) and xenografts grew in a manner that surrounded the sciatic nerve (Fig. 3f). The tumor weight indicated that transfection of DIAPH2-AS1 promoted the GC-NI of GC cells in vivo while silencing DIAPH2-AS1 exerted the reverse function (Fig. 3g).

Collectively, these data demonstrated that DIAPH2-AS1 promoted the metastasis and NI of GC in vivo.

### DIAPH2-AS1 promotes malignant behavior and NI potential of GC cells through NTN1 in vitro

**Fig. 4:**
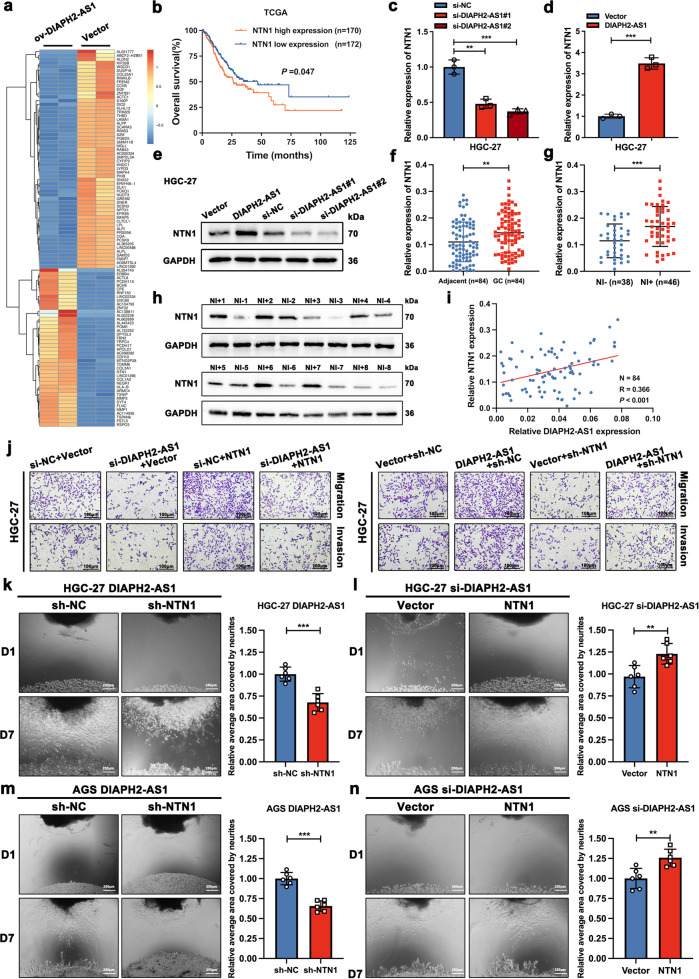
DIAPH2-AS1 promotes malignant behavior and NI potential of GC cells through NTN1 in vitro. **a** Heatmap exhibiting the DEGs in AGS cells transfected with DIAPH2-AS1 or not. **b** Kaplan–Meier survival analysis of OS of 172 GC patients with low expression of NTN1 versus 170 GC patients with high expression of NTN1 by log-rank (Mantel-Cox) test. **c**, **d** qRT-PCR analysis of NTN1 mRNA level in HGC-27 cells after transfection of si-DIAPH2-AS1 or DIAPH2-AS1. **e** Western blot analysis of NTN1 protein level in HGC-27 cells transfected with si-DIAPH2-AS1, DIAPH2-AS1, or corresponding control. **f** NTN1 mRNA level was quantified in 84 pairs GC and adjacent normal tissue via qRT-PCR. **g** NTN1 mRNA level was quantified in 46 NI-positive GC tissues compared with 38 NI-negative GC tissues via qRT-PCR. **h** Protein level of NTN1 was detected by western blot using 8 NI-positive GC tissues and 8 NI-negative GC tissues. **i** Correlation analysis was carried out to assess the correlation between the expression level of DIAPH2-AS1 and the mRNA level of NTN1 in 84 GC tissues. *N* = 84, *R* = 0.366, *P* < 0.001. **j** Transwell assay was performed utilizing indicated engineered HGC-27 cells. Scale bar: 100 μm. Data and error bars are shown as mean ± SD of triplicate independent replicate experiments and all data were analyzed by Student’s t test. **k**–**n** Representative D1 (day1) and D7 (day7) images of the DRG-GC cells co-culture model utilizing designated HGC-27 and AGS cells and corresponding statistic charts were shown. Scale bar: 250 μm. NI potential was measured according to the relative area covered by neurites calculated by ImageJ. Error bars represented the mean ± SD of six independent duplicate experiments and all data was analyzed by Student’s t test (*P < 0.05; **P < 0.01; ***P < 0.001).

To identify the downstream target via which DIAPH2-AS1 achieved its pro-metastatic malignant behavior and NI potential of GC cells, we performed the mRNA-seq analysis comparing the expression profile of HGC-27 cells transfected with DIAPH2-AS1 or not. A group of DEGs (*P* value < 0.05, log_2_FC > 2) possibly regulated by DIAPH2-AS1 was found, among which 324 genes were overexpressed and 783 genes were downregulated (Fig. 4a and Supplementary Fig. S3a). NTN1 attracted our attention among these DEGs for its extensive role in axonal guidance and cell migration [24]. More importantly, our previous works suggested that NTN1 promotes the progression and NI of GC [17–19]. Therefore, NTN1 was selected as the downstream target molecule of DIAPH2-AS1-mediated GC-NI for subsequent assays. Kaplan–Meier survival analysis from TCGA also indicated that GC patients with an increased expression of NTN1 were associated with a worse prognosis (Fig. 4b), and data from an online website (http://kmplot.com/analysis/) showed that the higher expression level of NTN1 predicts the shorter OS, FPS, and PPS (Supplementary Fig. S3b). Next, consistent with the result of mRNA-seq analysis, upregulation of DIAPH2-AS1 elevated the mRNA and protein expression of NTN1 in HGC-27 and AGS cells, and the opposite effect was observed after silencing DIAPH2-AS1 (Fig. 4c–e, and Supplementary Fig. S3c, d). Then, the expression level of NTN1 in GC samples was detected. Results showed that NTN1 was upregulated in 84 GC samples (Fig. 4f), and further elevated in 46 NI-positive GC samples than in 38 NI-negative GC samples (Fig. 4g). Western blot revealed that the protein level of NTN1 was also elevated in NI-positive GC tissues compared with NI-negative samples (Fig. 4h). We also found that the expression level of DIAPH2-AS1 was positively associated with mRNA level of NTN1 (Fig. 4i). Thus, we hypothesized that NTN1 likely acted as the downstream target of DIAPH2-AS1.

Subsequently, in vitro functional experiments, including wound-healing assay, Transwell assay, and DRG-GC cells co-culture model, were carried out to further verify our hypothesis. As the results showed, in the wound-healing assay (Supplementary Fig. S4a, b) and the Transwell assay (Fig. 4j, and Supplementary S4c–e), overexpression of NTN1 reversed the inhibitory effect on GC cells induced by si-DIAPH2-AS1, while transfection of sh-NTN1 significantly weakened the pro-metastasis phenotype of GC cells resulted from the overexpression of DIAPH2-AS1. Likewise, consistent results were observed in the DRG-GC cells co-culture model (Fig. 4k–n).

Through these data, we summarized that DIAPH2-AS1 promotes the migration, invasion, and NI potential of GC cells through NTN1 in vitro.

### DIAPH2-AS1 promotes metastatic behavior and NI potential of GC cells through NTN1 in vivo

**Fig. 5:**
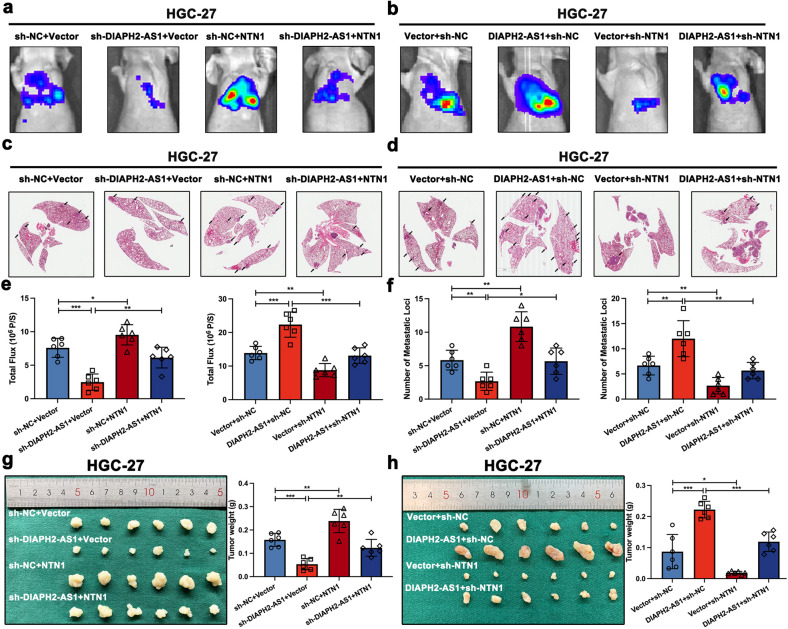
DIAPH2-AS1 promotes metastatic behavior and NI potential of GC cells through NTN1 in vivo. **a**–**f** Representative image of bioluminescent images, H&E staining of lung tissue (*n* = 6), and corresponding statistical charts were exhibited. **g**, **h** Xenograft neoplasms of the mouse sciatic nerve model and quantification of tumor weight of xenograft tumors were shown. Data and error bars were shown as mean ± SD of six independent replicate experiments and all data were analyzed by Student’s t test (*P < 0.05; **P < 0.01; ***P < 0.001).

In addition, the lung metastasis model and the mouse sciatic nerve model were also performed to further figure out whether NTN1 was indispensable for DIAPH2-AS1 to exert its oncogenic function in vivo. As the results showed, transfection of the overexpression plasmid of NTN1 in HGC-27 cells partially reversed the attenuated lesion of lung metastasis attributed to the silence of DIAPH2-AS1 (Fig. 5a, c, e). In addition, transfection of sh-NTN1 significantly rescued the lung metastasis enhanced by overexpressing DIAPH2-AS1 in HGC-27 cells (Fig. 5b, d, f). In vivo mouse sciatic nerve model also demonstrated that overexpression of NTN1 to a great extent offset the inhibitory effect of sh-DIAPH2-AS1 on the growth of tumor (Fig. 5g), and NTN1 knockdown repressed the tumor growth accelerated by DIAPH2-AS1 overexpression (Fig. 5h).

Thus, we confirmed that DIAPH2-AS1 promotes metastatic biological behavior and NI potential of GC cells through NTN1 in vivo.

### DIAPH2-AS1 upregulates NTN1 expression by interacting with NSUN2

**Fig. 6:**
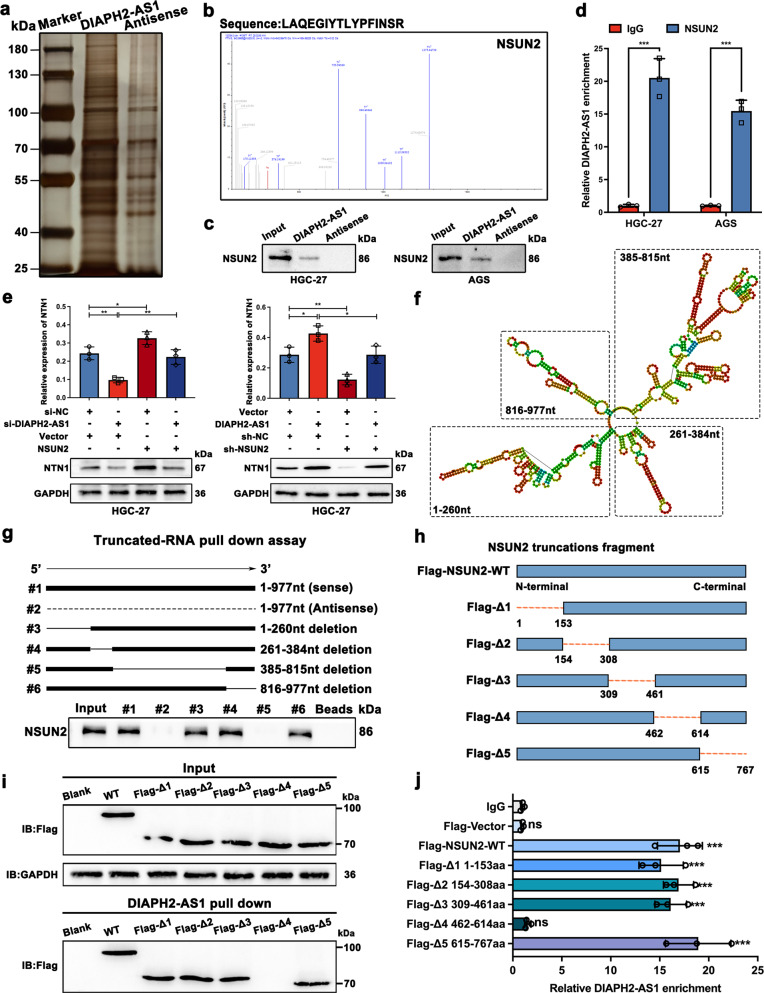
DIAPH2-AS1 upregulates NTN1 expression by interacting with NSUN2. **a** SDS-PAGE and silver staining using fractions pull downed by DIAPH2-AS1 and its antisense were performed using the lysis of HGC-27 cells. **b** The secondary mass spectrometry of NSUN2. **c** Western blot analysis confirmed that NSUN2 was enriched in the fractions pulled down by DIAPH2-AS1. **d** RIP assay verified that the antibody against NSUN2 could retrieve DIAPH2-AS1. **e** Relative mRNA and protein levels of NTN1 were detected by qRT-PCR and western blot in indicated HGC-27 cells. **f** The secondary structure of DIAPH2-AS1 was predicted by catRAPID (http://service.tartaglialab.com/). **g** The schematic diagram of the truncated mutant fragments of DIAPH2-AS1 (upper panel). Western blot analysis showed that the truncated fragment #5 of DIAPH2-AS1 was indispensable for the interaction between DIAPH2-AS1 and NSUN2 (bottom panel). **h** The diagrammatic sketch of FLAG-tagged WT or truncated mutant plasmids of NSUN2. **i** Western blot analysis indicated that the DIAPH2-AS1 failed to enrich NSUN2 after deleting the region of 462–614aa. **j** RIP assay showed that the fragment of NSUN2 without 462–614aa lost the binding capacity with DIAPH2-AS1. Data and error bars were shown as mean ± SD of triplicate independent replicate experiments and all data were analyzed by Student’s t test (*P < 0.05; **P < 0.01; ***P < 0.001).

Firstly, to test whether DIAPH2-AS1 functions as a competitive endogenous RNA (ceRNA), the pulldown assay was carried out to examine the association between DIAPH2-AS1 and Argonaute-2 (Ago2), the crucial component of RNA-induced silencing complex (RISC) which is indispensable for ceRNA network [25]. As the result showed (Supplementary Fig. S5a), Ago2 was not detected in the fractions pulled down by DIAPH2-AS1, which ruled out the ceRNA function of DIAPH2-AS1. Many studies suggested that interacting with RNA-binding proteins (RBPs) is also a prevalent way lncRNAs regulate the downstream biological factors. Thus, the eluted DIAPH2-AS1 pulldown fractions were resolved and then separated by SDS-PAGE and visualized by silver staining (Fig. 6a). Mass spectrometry analysis was further conducted to identify the potential downstream RBP of DIAPH2-AS1. The NOP2/Sun RNA methyltransferase 2 (NSUN2) (Fig. 6b), a critical m5C writer which is involved in the mRNA m5C modification and the progress of multitype tumors [26, 27], drew our attention. According to the TCGA database, the expression of NSUN2 was higher in GC patients’ tissues (Supplementary Fig. S5b), and the higher expression of NSUN2 also predicted a worse prognosis (Supplementary Fig. S5c). Therefore, we hypothesized that NSUN2 might serve as the key factor mediating the regulation of DIAPH2-AS1 on NTN1 by interacting with DIAPH2-AS1. Western blot analysis confirmed that NSUN2 was enriched in the fractions captured by DIAPH2-AS1 but not its antisense (Fig. 6c). We also conducted the RIP assay in combination with qRT-PCR analysis. A remarkable enrichment of DIAPH2-AS1 was detected by a specific antibody against NSUN2 compared with IgG (Fig. 6d). These findings confirmed that DIAPH2-AS1 could directly bind to NSUN2.

Furthermore, we found that si-DIAPH2-AS1 reduced the mRNA and protein levels of NTN1, but additional transfection of NSUN2 partially reversed the effect of si-DIAPH2-AS1 (Fig. 6e left panels, and Supplementary S5d). Analogously, transfection of sh-NSUN2 attenuated the DIAPH2-AS1-induced elevation of NTN1 mRNA and protein levels (Fig. 6e right panels, and Supplementary S5e), which demonstrated that DIAPH2-AS1 regulated NTN1 expression by recruiting NSUN2.

To figure out the specific binding region between DIAPH2-AS1 and NSUN2, we predicted the secondary structure of DIAPH2-AS1 by catRAPID (http://service.tartaglialab.com/) (Fig. 6f). Then, full-length or truncated DIAPH2-AS1 plasmids were constructed as schematic diagrams showed (Fig. 6g, upper panel) and subsequently, a mapping assay was conducted. The result showed that NSUN2 was detected in the fractions pulled down by wild type (WT) DIAPH2-AS1, truncated fragments #3, #4, and #6 but not antisense and truncated fragment #5 (Fig. 6g, lower panel). These data indicated that the 385nt–815nt of DIAPH2-AS1 was responsible for its interaction with NSUN2. Consistently, the prediction result of RBPsuite (http://www.csbio.sjtu.edu.cn/bioinf/RBPsuite/) also suggested that two fragments of DIAPH2-AS1 which possess a solid probability of interacting with NSUN2, also locate on the 385nt–815nt of DIAPH2-AS1 (Supplementary Fig. S5f). On the other hand, to identify which fragment of NSUN2 was fundamental for its binding with DIAPH2-AS1, we synthesized the FLAG-tagged WT plasmid of NSUN2 (WT) and the FLAG-tagged truncated mutant plasmids of NSUN2 Δ1 (1–153 amino acid deletion), Δ2 (154–308aa deletion), Δ3 (309–461aa deletion), Δ4 (462–614aa deletion) and Δ5 (615–767aa deletion) (Fig. 6h). Then the pulldown assay was performed using full-length DIAPH2-AS1, which demonstrated that DIAPH2-AS1 was unable to probe the truncated fragment of Δ4 (Fig. 6i). RIP assay also showed no association between the truncated fragment of Δ4 and DIAPH2-AS1, which further confirmed that 462–614aa region of NSUN2 was indispensable for their interaction (Fig. 6j).

Collectively, these results indicated that DIAPH2-AS1 enhanced the expression of NTN1 through interacting with NSUN2. The 385nt–815nt fragment of DIAPH2-AS1 accounted for the binding with the 462–614aa region of NSUN2.

### DIAPH2-AS1 stabilizes NSUN2 via attenuating ubiquitin-proteasome-mediated degradation

**Fig. 7:**
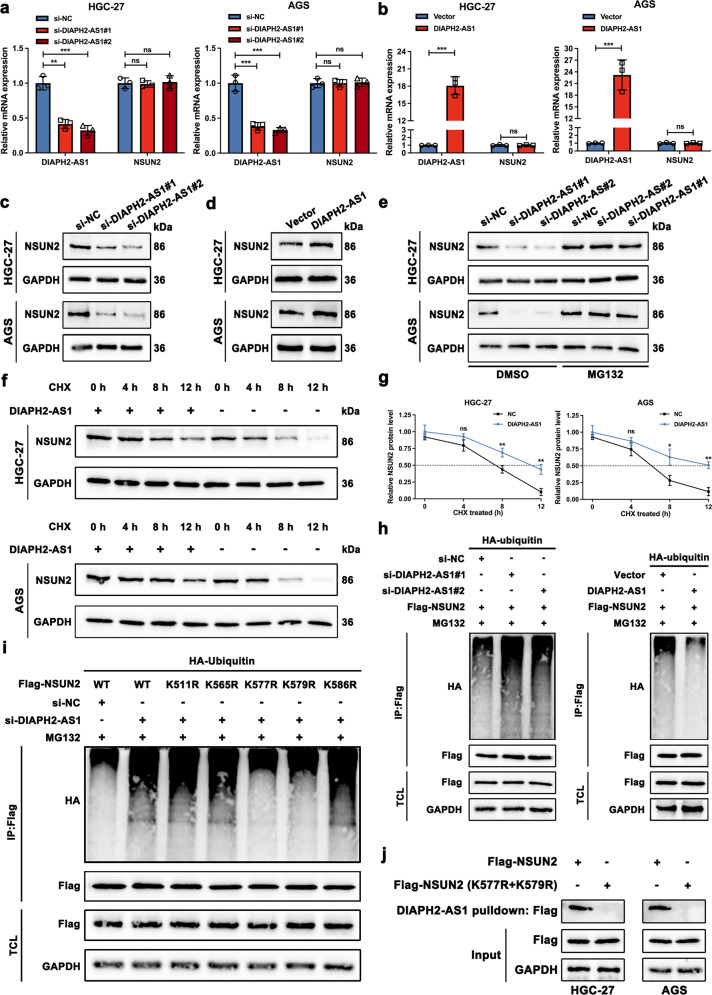
DIAPH2-AS1 stabilizes NSUN2 via attenuating ubiquitin-proteasome-mediated degradation. **a**, **b** Relative expression levels of DIAPH2-AS1 and NSUN2 were quantified by qRT-PCR in designated HGC-27 and AGS cells. Data and error bars were shown as mean ± SD of triplicate independent replicate experiments and all data were analyzed by Student’s t test (*P < 0.05; **P < 0.01; ***P < 0.001). **c**, **d** Western blot analysis was utilized to examine the protein level of NSUN2 using the aforementioned cells. **e** DIAPH2-AS1-knockdown and control HGC-27 and AGS cells were treated with MG132 (10 μmol/L) or DMSO for 10 h, and then western blot analysis was performed to measure the retaining protein level of NSUN2. **f** HGC-27 and AGS cells transfected with DIAPH2-AS1 or not were treated with CHX (50 μg/mL) for indicated periods. Then NSUN2 was detected by western blot. **g** Line charts of the relative protein level of NSUN2 of indicated periods. **h** The ubiquitination level of NSUN2 was examined in HGC-27 cells co-transfected with HA-tagged ubiquitin, FLAG-tagged NSUN2 plus si-DIAPH2-AS1, DIAPH2-AS1, or corresponding control. Designated HGC-27 cells were then subjected to MG132 (10 μmol/L) treatment for 10 h. Cell lysis was extracted to perform the co-immunoprecipitation (co-IP) assay which was carried out using anti-FLAG and examined by western blot via anti-HA. TCL: total cell lysate. **i** HGC-27 cells were transfected with plasmids expressing indicated FLAG-tagged WT or mutant NSUN2 plasmids (K511R, K565R, K577R, K579R, or K586R) and HA-tagged ubiquitin plasmids, along with specific si-RNA targeting DIAPH2-AS1 or not. Engineered HGC-27 cells were then subjected to MG132 (10 μmol/L) treatment for 10 h. co-IP and western blot analysis were performed as mentioned above to detect the ubiquitination level of WT and mutant FLAG-NSUN2. **j** HGC-27 and AGS cells were transfected with the plasmids of FLAG-tagged NSUN2 or FLAG-tagged mutant NSUN2 (K577R + K579R) and then a lncRNA pulldown assay was performed using DIAPH2-AS1 probe. Next western blot using the specific antibody against FLAG was conducted.

Previous studies reported that lncRNAs regulate their binding proteins in different manners, including acting as molecular scaffolds to provide a place for reactions and affecting the stability of their molecular chaperone [28, 29]. Therefore, we detected whether DIAPH2-AS1 affected the expression of NSUN2 first. qRT-PCR and western blot analyses showed that the mRNA expression of NSUN2 remained unchanged (Fig. 7a, b), while the protein level of NSUN2 altered markedly after DIAPH2-AS1 knockdown or overexpression in HGC-27 and AGS cells (Fig. 7c, d). These data suggested that DIAPH2-AS1 regulated NSUN2 expression at the posttranslational level.

Ubiquitination modification is a well-known posttranslational modification of proteins via which proteins will be degraded after they are marked by ubiquitin. It plays a decisive role in regulating numerous biological processes such as oncogenesis [30]. Thence, we tried to explore whether the interaction between DIAPH2-AS1 and NSUN2 regulated NSUN2 expression by the ubiquitin-proteasome pathway. As Fig. 7e showed, MG132, an inhibitor of the ubiquitin-proteasome pathway, attenuated si-DIAPH2-AS1-induced degradation of NSUN2 compared with the treatment of DMSO. In addition, the protein degradation assay was conducted by cycloheximide (CHX) treatment, an inhibitor blocking protein biosynthesis. We found that the overexpression of DIAPH2-AS1 inhibited the degradation of NSUN2 (Fig. 7f, g). These indicated that DIAPH2-AS1 might act as a protective factor on the stability of NSUN2. Next, the ubiquitination level of NSUN2 was detected by co-transfecting FLAG-tagged NSUN2 and HA-tagged ubiquitin into HGC-27 cells. Results indicated that the silence of DIAPH2-AS1 increased the ubiquitination level of NSUN2 and overexpressing DIAPH2-AS1 showed the opposite effects (Fig. 7h). Accordingly, we concluded that by binding with NSUN2, DIAPH2-AS1 strengthened the stability of NSUN2 via repressing the ubiquitin-proteasome pathway-mediated degradation.

As mentioned above, through mapping assay, we proved that DIAPH2-AS1 interacted with the 462–614aa fragment of NSUN2 via its region of 385–815nt. We next attempted to explain whether lysine sites within this interaction region participated in the ubiquitination of NSUN2. Online tools, including CPLM (http://cplm.biocuckoo.org/) and Phosphosite (https://www.phosphosite.org/) [31, 32], were utilized to predict the lysine sites located within the binding region. Five potential lysine sites (K511, K565, K577, K579, and K586) responsible for the ubiquitination of NSUN2 were obtained. Then, the WT and mutant NSUN2 plasmids were constructed, and the ubiquitination assay was performed. Mutations of K577 (K577R) and K579 (K579R) but not K511 (K511R), K565 (K565R), and K586 (K586R) weakened the ubiquitination level of NSUN2, which demonstrated that K577 and K579 were two crucial lysine sites mediating the ubiquitination-modification of NSUN2 (Fig. 7i). Finally, we also proved that mutations of K577 and K579 abolished the interaction between DIAPH2-AS1 and NSUN2 (Fig. 7j).

Therefore, according to these experiments, we summarized that DIAPH2-AS1 inhibited the ubiquitin-proteasome-mediated degradation of NSUN2 by binding with the 462–614aa fragment and masking the K577 and K579 of NSUN2.

### NSUN2-mediated m5C modification upregulates the expression of NTN1

**Fig. 8:**
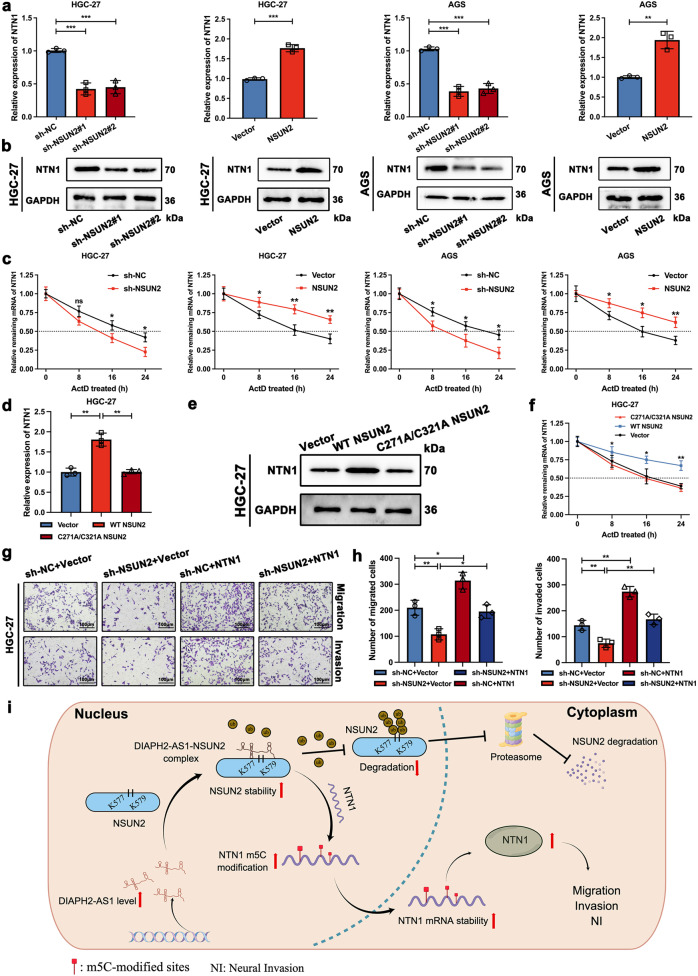
NSUN2-mediated m5C modification upregulates the expression of NTN1. **a**, **b** NTN1 mRNA and protein levels of designated HGC-27 and AGS cells were detected by qRT-PCR and western blot. **c** The degradation rate of NTN1 mRNA was calculated by qRT-PCR using indicated HGC-27 and AGS cells. Designated cells were treated with ActD (Actinomycin D, 2 mg/ml) for the indicated periods. **d**, **e** NTN1 mRNA and protein levels were detected by qRT-PCR and western blot in HGC-27 cells transfected with WT NSUN2, C271A/C321A NSUN2, or empty vector. **f** The degradation rate of NTN1 mRNA was measured utilizing HGC-27 cells transfected with WT NSUN2, C271A/C321A NSUN2, or empty vector as mentioned in (**d**) and (**e**). **g** Transwell assay including migration and invasion was performed utilizing indicated engineered HGC-27 cells. Scale bar: 100 μm. **h** Statistical graphs of the Transwell assay mentioned in (**g**) were exhibited. **i** The schematic diagram (created by Figdraw) depicting the mechanism by which the interaction between DIAPH2-AS1 and NSUN2 promotes GC-NI through NTN1 is shown. Data and error bars were shown as mean ± SD of triplicate independent replicate experiments and all data were analyzed by Student’s t test (*P < 0.05; **P < 0.01; ***P < 0.001).

It has been widely accepted that NSUN2 is a methyltransferase possessing the ability to catalyze the m5C modification of multiple RNAs such as tRNAs, mRNAs, and ncRNAs [33]. Therefore, we asked whether DIAPH2-AS1 influenced NTN1 expression in a manner of m5C modification mediated by NSUN2. As Fig. 8a, b showed, sh-NSUN2 decreased the expression of NTN1 mRNA and protein; on the contrary, NSUN2 upregulated both mRNA and protein levels of NTN1. In addition, the RNA half-life assay was performed, and the results proved that NSUN2 attenuated the degradation rate of NTN1 mRNA, and sh-NSUN2 shortened the half-life of NTN1 mRNA (Fig. 8c), which indicated that NSUN2 upregulated the mRNA and protein levels of NTN1 by modulating the mRNA stability of NTN1. Previous research had reported that NSUN2 altered the stability of target mRNA via its methyltransferase activity [34, 35]. Thus, to confirm whether NSUN2 maintained the stability of NTN1 mRNA via its activity of m5C modification, we constructed the C271A/C321A NSUN2 mutant plasmid, which lose the methyltransferase activity as previously reported [34, 35]. Compared with the WT NSUN2, C271A/C321A mutant NSUN2 elevated neither mRNA nor protein level of NTN1 (Fig. 8d, e and Supplementary Fig. S6a, b). Analogously, the RNA half-life assay also indicated that C271A/C321A mutant NSUN2 showed no apparent effects on the degradation rate of NTN1 mRNA (Fig. 8f and Supplementary Fig. S6c). Furthermore, a pulldown assay was conducted using HGC-27 and AGS cells transfected with the FLAG-tagged NSUN2 or FLAG-tagged C271A/C321A NSUN2 plasmid, which showed that the mutation of C271 and C321 of NSUN2 did not affect the interaction between DIAPH2-AS1 and mutant NSUN2 (Supplementary Fig. S6d). As a result, we confirmed that DIAPH2-AS1 upregulated the mRNA and protein levels of NTN1 with the aid of NSUN2, m5C modification activity of which strengthened the mRNA stability of NTN1.

Furthermore, function experiments were also performed to confirm whether NSUN2 affected the migration and invasion ability of GC cells through NTN1. Results of wound-healing and Transwell assays both showed that NTN1 overexpression rescued the inhibitory effect of sh-NSUN2 on the aggressive behavior of GC cells (Fig. 8g, h and Supplementary Fig. S7a, b, e, i, k), and NTN1 knockdown reversed the strengthened migration and invasion ability of GC cells induced by transfection of NSUN2 (Supplementary Fig. S7c, d, f, g, h, j, l).

In brief, we concluded that NSUN2 upregulated NTN1 in an m5C manner via which NSUN2 participated in the progress of DIAPH2-AS1-NTN1 axis-mediated GC-NI.

## Discussion

As a vital pathological feature, NI exists in multiple neoplasms, including pancreatic cancer, colon cancer, rectal cancer, and gastric cancer [2, 36–38]. NI was reported to be related to cancer-associated pain, tumor occurrence, and poor prognosis [36–39]. In GC, data from a previous meta-analysis from 13 studies containing 7004 GC patients revealed that the incidence of NI is 35.9%, and NI is significantly associated with a worse prognosis [2]. However, the underlying molecular mechanisms of GC-NI remain unclear.

Numerous research had elucidated the essential roles of lncRNAs in mediating the development and progression of multiple cancers [40]. In GC, lncRNA HOTAIR is overexpressed and promotes GC by acting as a ceRNA by sponging miR-331-3p [41], lncRNA EIF3J-DT enhances GC chemoresistance by autophagy activation [42], and hypoxia-induced lncRNA-CBSLR influences the progress of GC via regulating ferroptosis [43]. Furthermore, lncRNAs can also serve as the diagnostic and prognostic biomarkers for various cancers. For instance, lncRNA BLACAT1 could function as a non-specific diagnostic and prognostic biomarker for multiple cancers, including hepatocellular cancer, lung cancer, and breast cancer, due to its upregulated expression level in these cancers [44]. Although a great number of studies had made tremendous progress in clarifying the effects of lncRNAs on GC, the roles of lncRNAs in GC-NI remain unexplored.

Aiming to clarify the potential involvement of lncRNAs in GC-NI, firstly, we attempted to figure out the expression profile of lncRNAs in GC-NI by a transcriptome sequencing between 3 NI-positive and 3 NI-negative GC tissues. Combined with the verification in enlarged samples, we found that DIAPH2-AS1 was upregulated in NI-positive GC tissues and might function as the predictor factor for the diagnosis of GC-NI and the prognostic marker for GC patients. Subsequently, by the in vitro and in vivo experiments, we proved that DIAPH2-AS1 improved the migration, invasion, and NI capability of GC cells.

To explore how DIAPH2-AS1 conferred the invasive phenotype on GC cells, we performed an RNA sequencing by overexpressing DIAPH2-AS1 in GC cells. NTN1, the fundamental axon guidance factor, drew our attention among a group of differentially expressed genes. As the first discovered axon guidance molecule, NTN1 was initially considered to induce the growth and development of nerve axons [16, 24, 45, 46]. With the deepening of research, accumulating studies have revealed the critical functions of NTN1 in the progression of multiple cancers, including pancreatic cancer, colorectal cancer, and GC through its canonical receptors such as uncoordinated 5A-D (UNC5A-D), deleted in colorectal cancer (DCC), neogenin, and down syndrome cell adhesion molecule (DSCAM) [19, 22, 47–50]. More importantly, our previous works revealed the involvement of NTN1 in GC-NI [19, 22]. Therefore, we speculated that the promoting effects of DIAPH2-AS1 on GC-NI relied on the modulation of NTN1, which was further confirmed by the following experiments.

Next, we attempted to figure out how DIAPH2-AS1 regulated NTN1. Previous studies revealed that the prevalent mechanisms of how lncRNAs influence their downstream factors include functioning as ceRNAs, cis-regulation or trans-regulation, modulating the subcellular location of mRNAs, and interacting with RBPs [29]. Interaction of lncRNAs with RBPs has always been the study hotspot as interaction with RBPs gives more diverse biological effects to lncRNAs [51]. Thus, we conducted the lncRNA pulldown assay and MS. Considered for the cancer-promoting effect and an essential role in m5C modification [35, 52], we focused on NSUN2, which might mediate the regulation of DIAPH2-AS1 on the expression of NTN1. As an RNA-binding protein belonging to NOL1/NOP2/SUN (NSUN) domain family, NSUN2 is found to regulate the methylation that transforms cytosine into 5-methylcytosine (m5C) in a variety of RNAs such as tRNAs, mRNAs and ncRNAs [33], which is involved in the progress of multitype tumors such as GC and bladder cancer [26, 35, 53]. Pulldown and RIP assays both validated the interaction of DIAPH2-AS1 with NSUN2. Following rescue experiments confirmed that via the interaction with NSUN2, DIAPH2-AS1 modulated both the mRNA and protein levels of NTN1.

Next, we questioned how DIAPH2-AS1 modulated the expression of NTN1 through binding with NSUN2. Studies revealed that the regulatory effects of lncRNAs on downstream molecules depend mainly on the function of their binding RBPs. LncRNA KB-1980E6.3 recruited IGF2BP1 and further facilitated the mRNA stability of c-myc [54]. LncRNA CASC9 interacts with CPB and then elevates LAMC2 expression by regulating the modification of histone acetylation [55]. It was reported that NSUN2 promotes the pathogenesis of diverse malignancies by stabilizing mRNAs by catalyzing the m5C modification [34, 35]. Thus, we supposed that NSUN2 upregulated the mRNA and protein expression levels of NTN1 by facilitating the mRNA stability of NTN1 through m5C modification, which was confirmed by the following experiments. Our work revealed a novel role of posttranscriptional m5C modification in GC-NI. However, the specific m5C-modified motif of NTN1 was unexplored. In one of our ongoing studies, we performed methylated RIP sequencing (MeRIP Seq). We found the enrichment of m5C modification in the 3’-untranslated region (3’-UTR) and coding sequence (CDS) of NTN1 mRNA in GC, suggesting its worth for further exploration in GC-NI.

Increasing evidence suggests that lncRNAs exert their functions on RBPs in various ways, such as adjusting protein stability [56], affecting the phosphorylation level [57], or competitively binding with the substrate of RBPs [58]. Among these mechanisms, that lncRNAs regulate the stability of RBPs is one of the essential manners. As reported previously, lncRNA PVIT prevents p-STAT3 from ubiquitin-proteasomal degradation and promotes angiogenesis by facilitating the STAT3/VEGFA pathway [59]. LncRNA MNX1-AS1 speeds up the progression of colorectal cancer by protecting YB1 from ubiquitination modification-mediated degradation [60]. In our work, mRNA expression of NSUN2 didn’t change in response to the alteration of DIAPH2-AS1, whereas DIAPH2-AS1 significantly increased the protein level of NSUN2. Furthermore, with the treatment of MG132 and CHX, we proved that DIAPH2-AS1 increased the stability of NSUN2 via attenuating the ubiquitination-proteasomal degradation, therefore enhancing the latter’s function of m5C modification and elevating NTN1 expression.

Accumulating studies have revealed that the concrete regulation of lncRNAs on RBPs largely depends on their specific binding region. Wen Ni et al. reported that lncRNA GAS5 binds with the WW domain of YAP and facilitates the S127 phosphorylation of YAP, thereby inducing the degradation of YAP via the ubiquitin-proteasome pathway [28]. Thence, the mapping assay was implemented to further elucidate the definite binding region. Pulldown assay utilizing truncated fragments of DIAPH2-AS1 or NSUN2 suggested that DIAPH2-AS1 was directly bound to the 462–614aa region of NSUN2 via its internal region of 385–815nt. A previous study reported that through masking K139 located in the binding area, lncRNA LINRIS enhanced the stability of IGF2BP2 by weakening the ubiquitination level of IGF2BP2 [61]. Therefore, as we had proved that DIAPH2-AS1 maintained the protein level of NSUN2 by attenuating the ubiquitin-proteasomal degradation, we further identified the functional lysine sites within the binding region of NSUN2. The potential ubiquitination-modified lysine sites within the binding region were further predicted using CPLM and Phosphosite, including K511, K565, K577, K579, and K586. Further results indicated that the ubiquitination level of NSUN2 was decreased upon the mutation of lysine sites of K577 and K579. In addition, K577R and K579R also abolished the interaction between DIAPH2-AS1 and NSUN2. Therefore, we confirmed that by masking two functional lysine sites (K577 and K579), DIAPH2-AS1 prevented NSUN2 from ubiquitin-proteasome pathway-mediated degradation.

## Conclusion

This work identified the upregulation of DIAPH2-AS1 in NI-positive GC, which may serve as a diagnosis factor for NI-positive GC. DIAPH2-AS1 enhances the migration, invasion, and NI potential of GC cells via modulating the expression of NTN1. DIAPH2-AS1 interacts with NSUN2 and protects NSUN2 from ubiquitin-proteasome system-mediated degradation, which strengthens the mRNA stability of NTN1 in an m5C modification-dependent manner (Fig. 8i). Our work may shed light on elucidating the biogenesis of NI-positive GC and the development of novel diagnostic and therapeutic targets of GC-NI.

## Materials and methods

### Clinical samples

Tissue samples used in this study were gathered from GC patients who underwent radical gastrectomy at the Department of Gastric Surgery, the First Affiliated Hospital of Nanjing Medical University from November 2014 to September 2015. This study was approved by the Ethics Committee of the First Affiliated Hospital of Nanjing Medical University. All patients signed written informed consent.

### Cell culture

The immortalized normal human gastric mucosa epithelial cell GES-1 and human GC cell lines, including HGC-27, AGS, KATO-III, MKN-45, MKN-28, and NCI-N87, were all purchased from Shanghai Institutes for Biological Sciences. AGS cells were cultured with F12K medium (WISENT, Canada), and KATO-III were cultured in Dulbecco’s modified Eagle’s medium (DMEM H-21 4.5 g/Liter Glucose, WISENT, Canada). In contrast, other cells were cultured with RPMI 1640 medium (WISENT, Canada). All media were supplemented with 10% bovine serum (WISENT, Canada) and 1% penicillin/streptomycin (Gibco, CA, USA). All cell lines were grown in a humidified cell incubator (37 °C, 5% CO_2_).

### RNA preparation and quantitative real-time polymerase chain reaction (qRT-PCR)

According to the instructions, TRIzol reagent (Invitrogen, Carlsbad, CA, USA) was utilized to purify the total RNA of tissues or cells. The fractions of the nucleus and cytoplasm were separated by NE-PER Nuclear and Cytoplasmic Extraction Reagents (Thermo Fisher Scientific, MA, USA). The quality and concentration of purified RNA were measured by NanoDrop ND-2000 spectrophotometer (Thermo Fisher Scientific, MA, USA). 1 μg RNA was reversely transcribed to cDNA via a TRUE script RT Kit (Proteinbio, Nanjing, China). Then qRT-PCR was conducted using 2× Universal SYBR Green qPCR Supermix (Proteinbio, Nanjing, China) with a 7500 Real-Time PCR System (Applied Biosystems, Waltham, MA, USA). Expression of GAPDH and small nuclear U6 were utilized as internal references for lncRNAs and mRNAs localized in cytoplasm and nucleus, respectively. We purchased all primers from RiboBio (Guangzhou, China), and sequences were written down in Table S1 (Supplementary Table S1).

### RNA stability assay

To measure the stability of NTN1 mRNA, HGC-27 and AGS cells were treated with 2 mg/ml actinomycin D (Sigma-Aldrich, St. Louis, MO, USA) and then we extracted the total RNA of HGC-27 and AGS cells at indicated periods (0 h, 8 h, 16 h, 24 h). qRT-PCR was conducted to calculate the relative remaining NTN1 mRNA level.

### Transfection

To establish the stable DIAPH2-AS1-overexpression and DIAPH2-AS1-knockdown GC cell lines, the human DIAPH2-AS1 overexpression plasmid and lentivirus-sh-DIAPH2-AS1 were bought from GeneChem (Shanghai, China). DIAPH2-AS1 specific small interference RNAs (siRNAs) were chemically synthesized by RiboBio (Guangzhou, China), and related sequences are recorded in Table S2 (Supplementary Table S2). Transfection was strictly performed in accordance with the manufacturer’s protocol of Lipofectamine3000. Stably transfected cell lines were selected utilizing puromycin.

### Western blot and immunoprecipitation

Using RIPA lysis buffer, we purified the total protein of GC tissues and cells. Via a BCA protein assay kit (Leagene Biotechnology, Beijing, China), protein concentration was detected. Through SDS-PAGEs, lysates of tissue samples or GC cells were separated and then transferred onto polyvinylidene difluoride (PVDF) membranes. After blocking the membranes by the treatment of 5% evaporated milk for 1 h at normal room temperature, then the membranes were incubated with primary antibodies mentioned in supplementary table S3 at 4 °C for about 12 h. Next, using TBST buffer, the membranes were washed 3 times and incubated with HRP-conjugated secondary antibodies for 2 h at normal room temperature. Finally, the blots on the membranes were exposed using ECL chemiluminescent reagents (Millipore, MA, USA) by a BioSpectrum 600 Imaging System (Thermo Fisher, MA, USA).

For immunoprecipitation, in brief, precooled PBS was used to wash GC cells, and then NP-40 lysis solution (Beyotime, Shanghai, China) was used to extract the total proteins of GC cells. Cell lysates were immunoprecipitated with the indicated antibodies after pre-clearing with Protein A/G PLUS-Agarose (SC-2003, Santa Cruz Biotechnology, CA, USA). The host species-derived purified immunoglobulin G (IgG) (12370, Sigma-Aldrich, St. Louis, MO, USA) was used as a non-specific control. Immunocomplexes were gained using Protein A/G PLUS-Agarose. Immunoprecipitated proteins were mixed with SDS-PAGE loading buffer and then eluted by boiling, subsequently detected by western blotting. Antibodies used in this study were listed in Table S3 (Supplementary Table S3).

### Transwell assay

To measure the migration and invasion capability of GC cells, the chambers (Corning, NY, USA) supplemented with Matrigel (BD Biosciences, New Jersey, USA) or not in the upper chamber were used. The chamber size is suitable for placing in the plastic 24-well plate. After 800 μL medium mixed with 10% FBS was added in the bottom chamber, HGC-27 (3 × 10^4^) and AGS (3 × 10^4^) cells suspended in 200 μL serum-free medium were added in the upper chamber. Incubating for 48 h, the culture medium was poured, and GC cells adhered to the membrane were fixed with paraformaldehyde and stained with 0.1% crystal violet. After wiping cells with cotton in the upper chamber, remaining GC cells adhered to the membrane of the bottom chamber were observed by microscope.

### Wound-healing assay

HGC-27 and AGS cells were planted in the plastic 6-well plate and cultured under 37 °C. When GC cells overgrew the bottom of the plate, sterile 200 μL pipet tips were utilized to create a thin scratch in each well. The scratch was marked so that the same field could be positioned every time. The scratches were recorded at 0 h and 48 h, respectively, to calculate the migration rates by an inverted microscope (Olympus Optical Co., Ltd., Tokyo, Japan). We refreshed the culture medium before photographing and cleaned cells 3 times to wash the cellular debris using 2 ml PBS.

### DRG-GC cells co-culture model

Based on the previously described method [22], we conducted a refined in vitro dorsal root ganglion (DRG)-GC cells co-culture model. In brief, being suspended in 25 μL growth-factor-reduced Matrigel (BD Biosciences, New Jersey, USA), 2 × 10^5^ GC cells were placed in the center of the 12-well plates. DRGs gained from 8-day-old Wistar rats were placed about 1 mm away from the Matrigel mixed with GC cells and fixed by 25 μL Matrigel. To eliminate the possibility of non-specific attraction of GC cells, we positioned an additional 25 μL Matrigel without GC cells on the opposite side. The GC cells and DRGs were incubated in Neurobasal Medium (Gibco, CA, USA) supplemented with 10% FBS (WISENT, Canada), 1% antibiotic containing penicillin and streptomycin (Gibco, CA, USA), 2% B-27 (Gibco, CA, USA), and 0.5 mM L-glutamine (Gibco, CA, USA). The medium was refreshed every 2 days. Immunofluorescence was performed using PGP 9.5, a specific neuronal marker to authenticate the DRG tissues.

### In vivo NI model

Nanjing Medical University Ethics Committee approved the animal studies. We purchased four-week-old BALB/c nude mice from the Department of Laboratory Animal Center of Nanjing Medical University. Mice were randomly divided into indicated groups. According to the previously reported technique [22], we exposed the sciatic nerve surgically under anesthesia, maintaining isoflurane inhalational isoflurane. Using a 25-μL Hamilton syringe (Hamilton Bonatus Co., Ltd, Switzerland), 5 μL PBS containing 2 × 10^5^ GC cells was slowly injected into the periphery of the sciatic nerve. Considering the hindlimb paw muscles were innervated by the sciatic nerve, the function of the sciatic nerve was assessed by the paw span calculated by the distance between the first and fifth toes of the hind limbs after 4 weeks. The mice were subsequently sacrificed and next, we collected and weighed xenografts. The tumor weight and hindlimb function were used as indicators to assess the severity of NI.

### RNA pulldown assay

Expression vectors for full-length DIAPH2-AS1 and its truncated fragments were synthesized and transcribed using the T7 RNA polymerase (Promega, Madison, Wisconsin, USA) in vitro. After in vitro transcription, DIAPH2-AS1 and truncated fragments were biotinylated using a Biotin RNA Labeling Mix kit (Roche, Basel, Switzerland, USA). Then the pulldown assay was conducted with a Pierce Magnetic RNA-Protein Pull-Down Kit (Thermo Fisher Scientific, Waltham, MA, USA). Biotin-labeled RNA was incubated with streptavidin magnetic beads at room temperature for 1 h. Total cell lysates were prepared freshly and added Protease/Phosphatase Inhibitor Cocktail and RNase inhibitors in each step of the reactions. Probes-beads complexes were incubated with cell lysates at 4 °C for 6 h. After thoroughly washing to remove unbound proteins, RNA-protein binding complexes were added to SDS buffer and boiled. Subsequently, eluted proteins were observed by silver staining and detected using western blot and Mass Spectrometry analysis.

### RNA immunoprecipitation (RIP) assay

According to the instruction manual, RIP assay was performed using the EZ-Magna RIP Kit (Millipore, Bedford, MA, USA). Approximately 2 × 10^7^ HGC-27 and AGS cells were lysed with 100 μL RIP lysis buffer for one immunoprecipitation. 5 μg antibody against target protein or corresponding IgG were incubated with Protein A/G Magnetic Beads at room temperature for half an hour. Then, 100 μL RIP lysates were mixed with the beads-antibody complex in a RIP Immunoprecipitation Buffer and incubated at 4 °C for 12 h. Finally, captured RNA was detected and analyzed by qRT-PCR.

### Animal experiment of in vivo metastasis assay

Animal experiments in our study were approved by the Nanjing Medical University Ethics Committee. Department of Laboratory Animal Center of Nanjing Medical University provided us with four-week-old BALB/c nude mice. Mice were randomly divided into indicated groups.

In vivo metastasis assay was performed by establishing a lung metastasis model. Briefly, HGC-27 cells labeled with luciferase (1 × 10^6^ cells per 100 μL PBS) transfected with indicated vectors were injected into the tail veins of mice. After five weeks, an abdominal injection of D-luciferin sodium salt stock solution (150 mg/ml) was used to detect the metastatic lung lesion after treating mice with 2% isoflurane. Then bioluminescent signals were measured via the IVIS Spectrum Xenogen Imaging System (Caliper Life Sciences, Hopkinton, MA, USA). After IVIS, mice were sacrificed, and their lungs were collected for HE staining to quantify the metastatic loci by a microscope.

### Hematoxylin and eosin (H&E) staining of tissues

Initially, the samples were placed in alcohol for hydration and then mounted using microscope slides. Next, to hydrate the tissue samples, we stirred the slide with deionized water for half a minute. Subsequently, the slides were placed in a bottle containing hematoxylin and stirred for half a minute, then washed with deionized water for 30 s. Slides were stained with 1% eosin and samples were hydrated with 95% and 100% alcohol sequentially. Finally, the alcohol was removed with xylene. Results were observed by a microscope.

### IHC analysis

The collected tumor samples were fixed with 4% paraformaldehyde immediately after removal from the body and embedded in paraffin. The sections were then incubated overnight at 4 °C in the refrigerator with the specific primary antibody of PGP9.5. On the second day, the sections were washed twice in PBS and incubated for 1 h at room temperature with HRP-polymer-conjugated secondary antibodies (Abcam, UK). Afterward, the sections were stained using 3,3-diaminobenzidine solution and haematoxylin. Finally, the slides were observed under a microscope (Olympus, Tokyo, Japan) and images were captured. Three senior pathologists independently determined whether the GC tissue was NI-positive or NI-negative based on the definition of NI mentioned in the introduction section, with a representative image of NI-positive GC tissue shown in Supplementary Fig. S1a. Information about the primary antibody can be found in Supplementary Table S2.

### Statistical analysis

SPSS 26.0 (IBM, SPSS, Chicago, IL, USA) and GraphPad Prism, version 9.00 (GraphPad Software, CA, USA) were utilized to conduct statistical analyses. Results were exhibited as the mean ± standard deviation (SD). The statistical significance of the two groups was calculated utilizing the Student’s t-test. The association between the expression of DIPAH2-AS1 and clinicopathological parameters was calculated by Chi-square test. The correlation between expression of DIAPH2-AS1 and that of NTN1 was tested by Pearson’s correlation analysis. Overall survival (OS), first progression survival (FPS), and post-progression survival (PPS) were analyzed by log-rank test. *P* < 0.05 was regarded as statistically significant.

## Supplementary information

Supplementary Materials

Supplementary Table S4 NInegative--NIpositive.all.gene

aj-checklist

## Data Availability

The datasets supporting the conclusions of this article are included within the article and its Additional files.
