# Supplementary material for: Long noncoding RNA DIAPH2-AS1 promotes neural invasion of gastric cancer via stabilizing NSUN2 to enhance the m5C modification of NTN1
Source: Cell Death Dis. 2023 Apr 10;14(4):260. doi: 10.1038/s41419-023-05781-5 (PMC10086070; doi:10.1038/s41419-023-05781-5)
Supplement: Supplementary file 1 — Supplementary Materials [file 41419_2023_5781_MOESM1_ESM.docx]

**Supplementary Materials**

**Supplementary Figure S1**


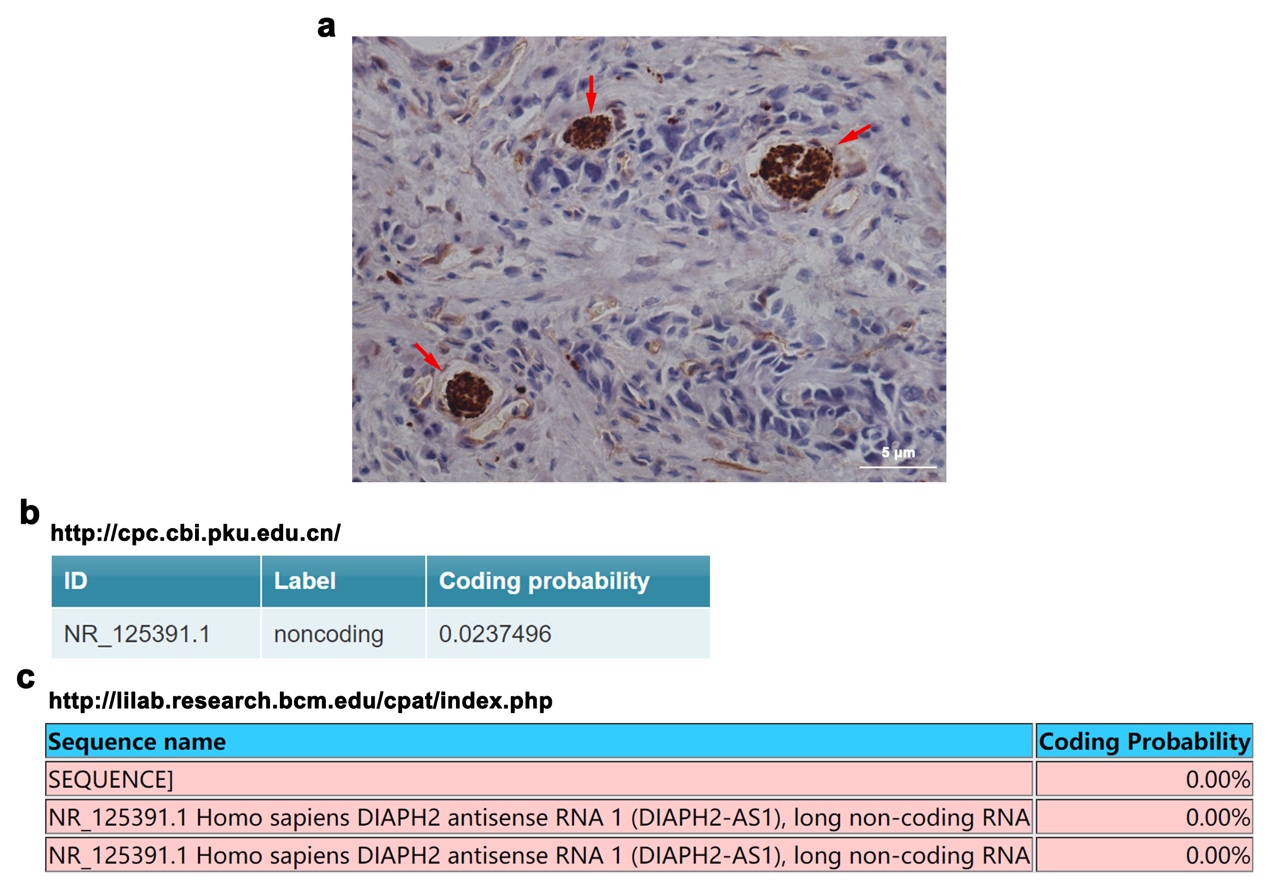


**a** The representative image of IHC staining of NI-positive GC tissue using PGP9.5 specific antibody. Red arrows represent nerves. Scale bar: 5μm. **b, c** The predicting results of the coding potential of DIAPH2-AS1 using two online tools including Coding Potential Calculator (CPC) (http://cpc.cbi.pku.edu.cn/) and RNA coding potential assessment tool (CPAT) (http://lilab.research.bcm.edu/cpat/index.php).

**Supplementary Figure. S2**


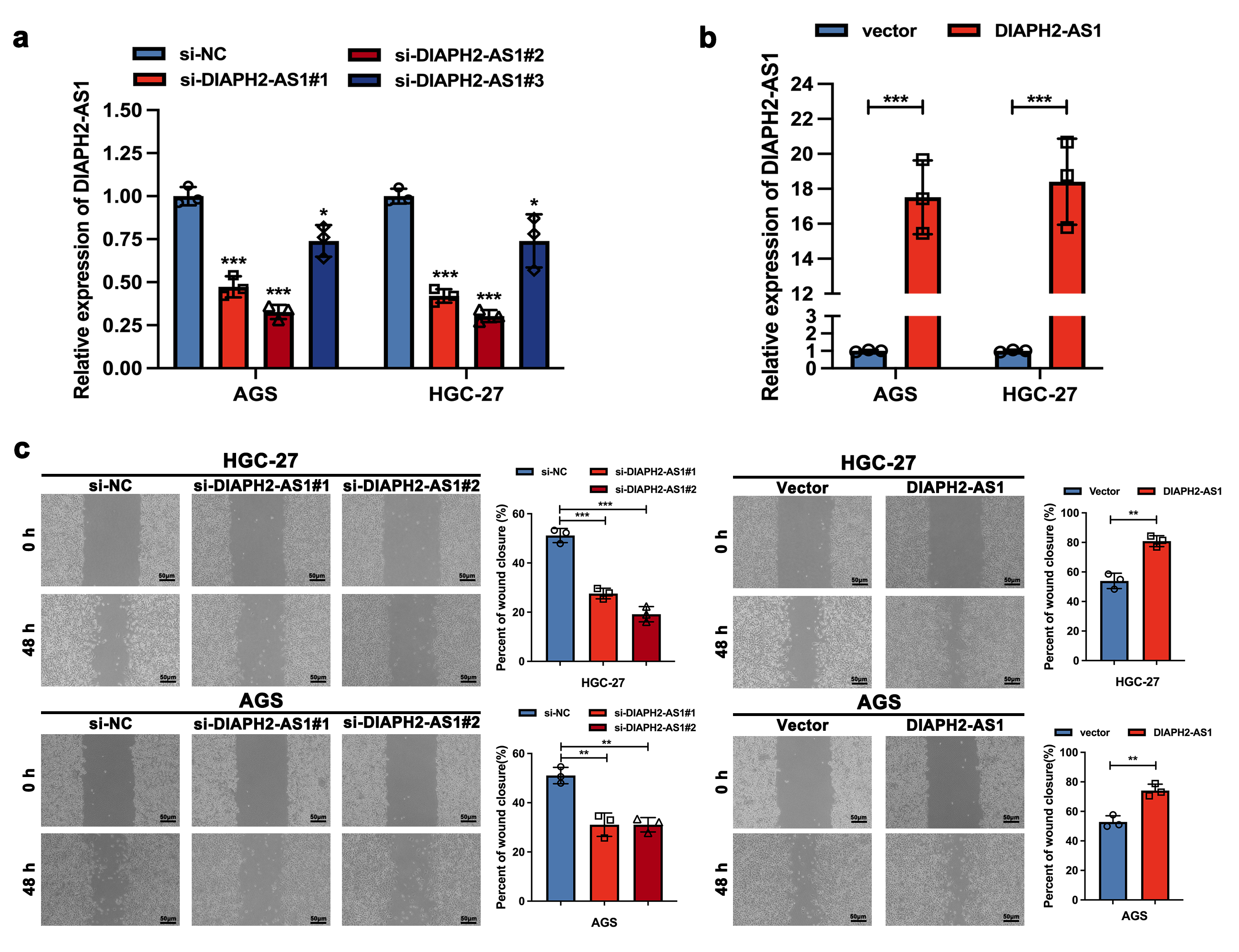


**a, b** Relative expression of DIAPH2-AS1 was quantified by qRT-PCR in HGC-27 and AGS cells transfected with indicated si-RNAs or plasmids to verify the transfection efficiency. **c** The wound healing assay was performed using designated HGC-27 and AGS cells. Scale bar: 50μm. Corresponding statistical graphs were shown. Data and error bars were shown as mean ± SD of triplicate independent replicate experiments and all data were analyzed by Student's t test (*P < 0.05; **P < 0.01; ***P < 0.001).

**Supplementary Figure. S3**


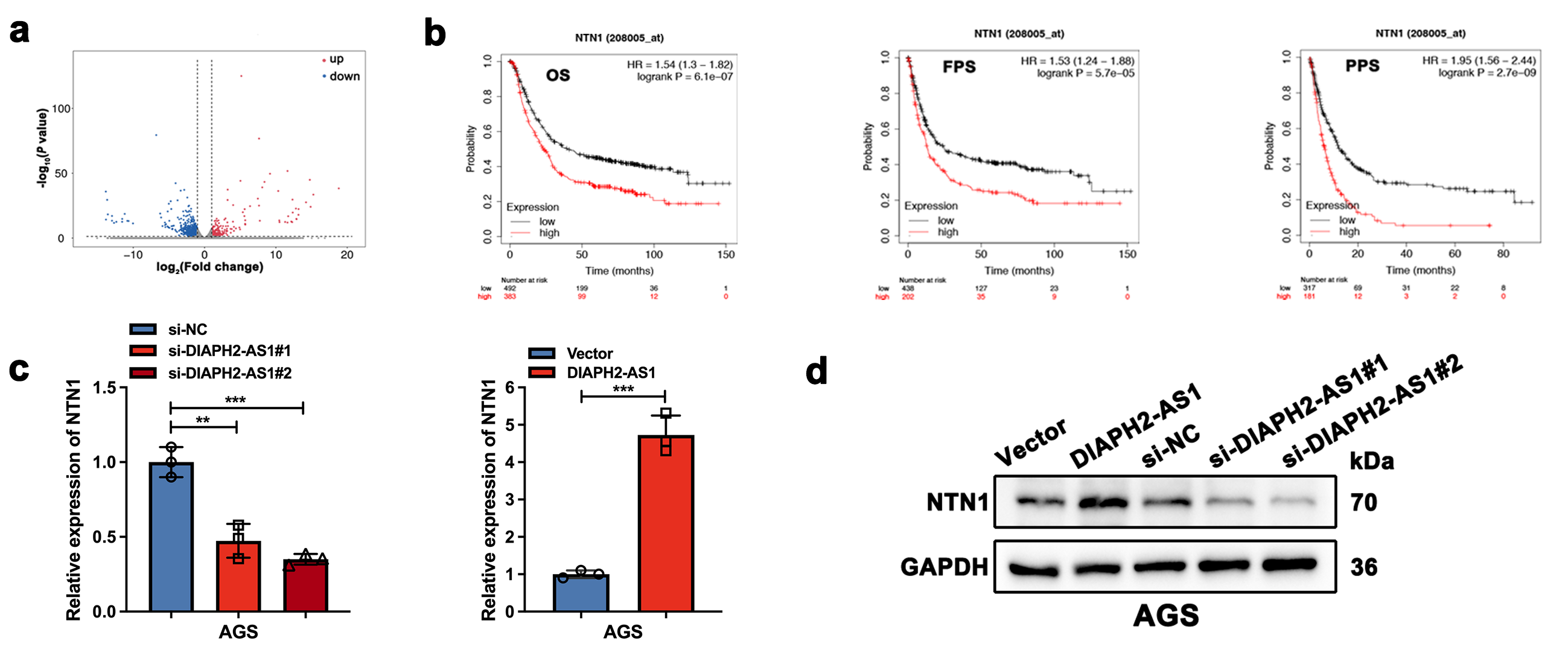


**a** Volcano plot exhibiting the DEGs in DIAPH2-AS1-overexpressed AGS cells compared with control AGS cells. **b** Kaplan-Meier survival analysis of overall survival (OS), first progression survival (FPS), and post progression survival (PPS) using public data from an online database (KM plotter: http://kmplot.com/analysis/) comparing patients with low NTN1 expression and patients with high NTN1 expression by log-rank (Mantel-Cox) test. **c** qRT-PCR analysis of NTN1 mRNA level using indicated AGS cells. **d** Western blot analysis of NTN1 protein level in designated AGS cells. Data and error bars were shown as mean ± SD of triplicate independent replicate experiments and all data were analyzed by Student's t test. (*P < 0.05; **P < 0.01; ***P < 0.001).

**Supplementary Figure. S4**


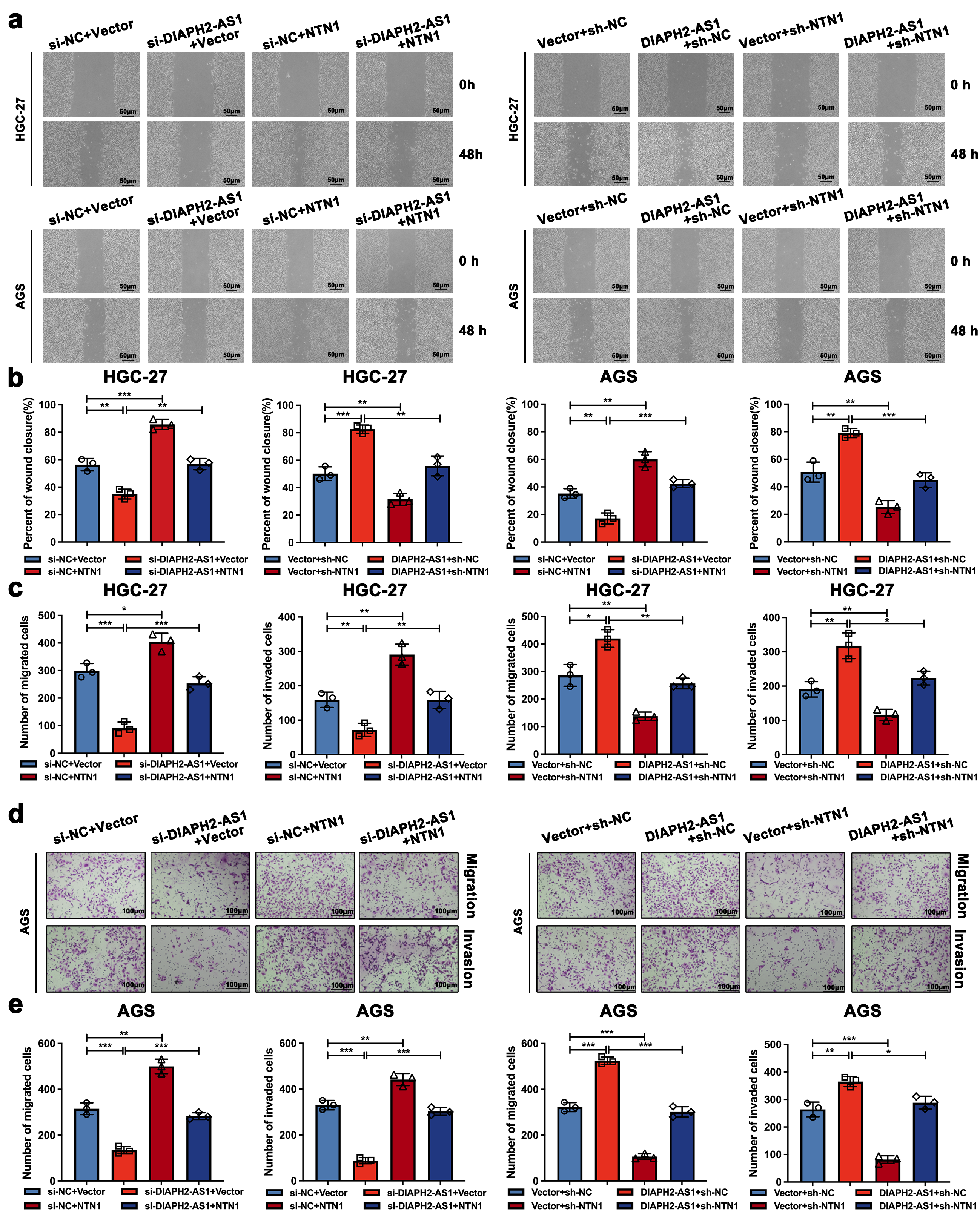


**a** Representative images of wound healing assay using indicated engineered HGC-27 and AGS cells. Scale bar: 50μm. **b** Quantification of the migration distance of designated HGC-27 and AGS cells are displayed. **c** Statistical graphs of Transwell assay of indicated HGC-27 cells. **d** Transwell assay was performed utilizing indicated engineered AGS cells. Scale bar: 100μm. **e** Statistical graphs of the Transwell assay using indicated AGS cells were shown. Data and error bars were shown as mean ± SD of triplicate independent replicate experiments and all data were analyzed by Student's t test. (*P < 0.05; **P < 0.01; ***P < 0.001).

**Supplementary Figure. S5**


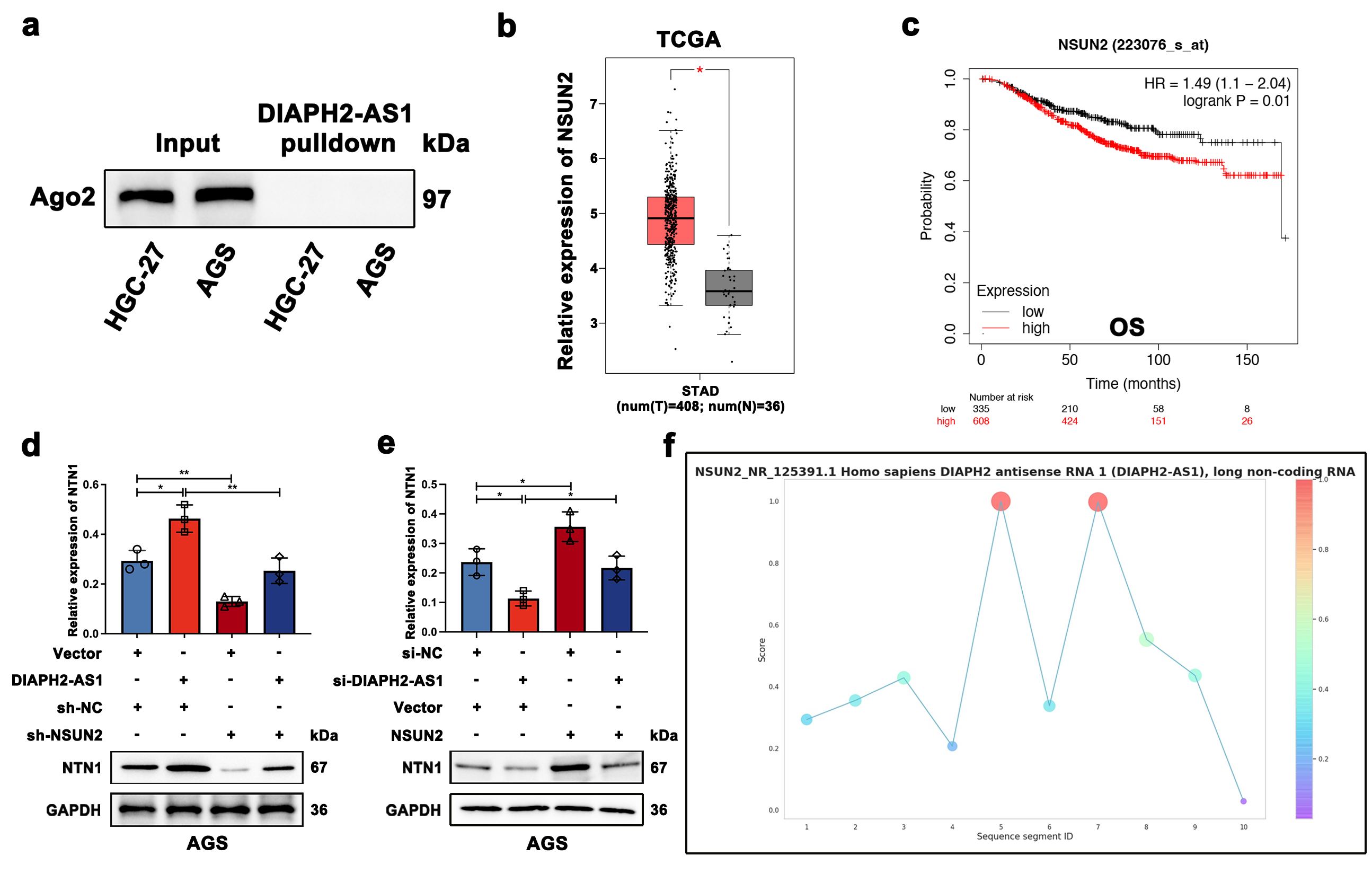


**a** Pulldown assay using DIAPH2-AS1 and its antisense plus western blot were performed to detect Ago2 using lysates of HGC and AGS cells. **b** Relative expression of NSUN2 of GC tissue (T) and normal tissue (N) from TCGA database. **c** OS of GC patients with high or low expression of NSUN2 were analyzed by online tools (http://kmplot.com/analysis/). **d, e** Relative mRNA and protein levels of NTN1 were detected by qRT-PCR and western blot in AGS cells transfected with indicated plasmids. Data and error bars were shown as mean ± SD of triplicate independent replicate experiments and all data were analyzed by Student's t test. (*P < 0.05; **P < 0.01; ***P < 0.001). **f** The potential binding region located on DIAPH2-AS1 for its interaction with NSUN2 was predicted by RBPsuite (http://www.csbio.sjtu.edu.cn/bioinf/RBPsuite/).

**Supplementary Figure. S6**

**
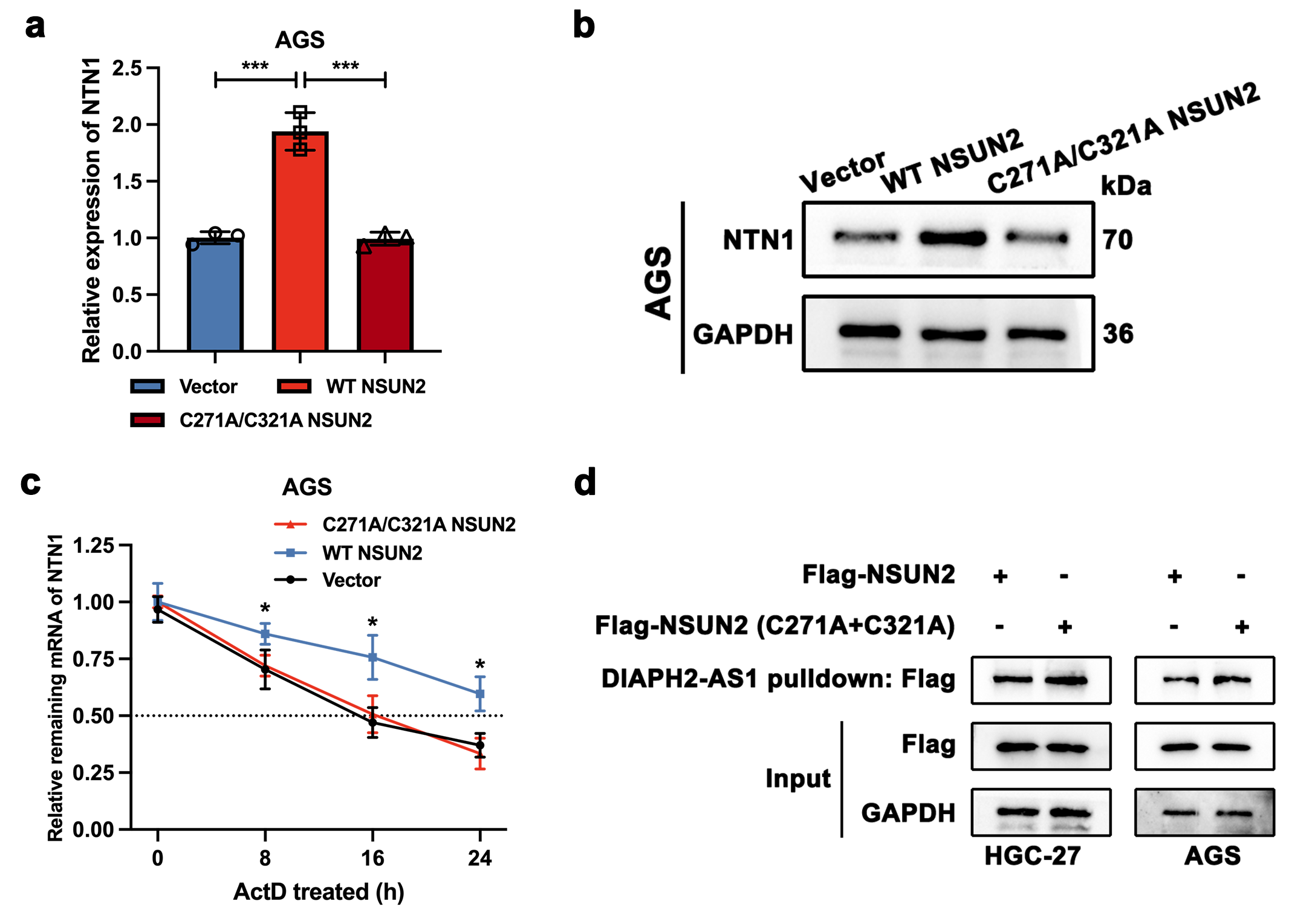
**

**a, b** NTN1 mRNA and protein levels were detected by qRT-PCR and western blot in AGS cells transfected with WT NSUN2, C271A/C321A NSUN2, or empty vector. **c** The degradation rate of NTN1 mRNA was measured utilizing AGS cells transfected with WT NSUN2, C271A/C321A NSUN2, or empty vector as mentioned in **a** and **b**. **d** HGC-27 and AGS cells were transfected with the plasmid of FLAG-tagged NSUN2 or FLAG-tagged C271A/C321A NSUN2. Pulldown assay was then performed as previously described, followed by a western blot analysis that confirmed that the mutant NSUN2 was also present in the fractions pulled down by DIAPH2-AS1. Data and error bars were shown as mean ± SD of triplicate independent replicate experiments and all data were analyzed by Student's t test. (*P < 0.05; **P < 0.01; ***P < 0.001).

**Supplementary Figure. S7**


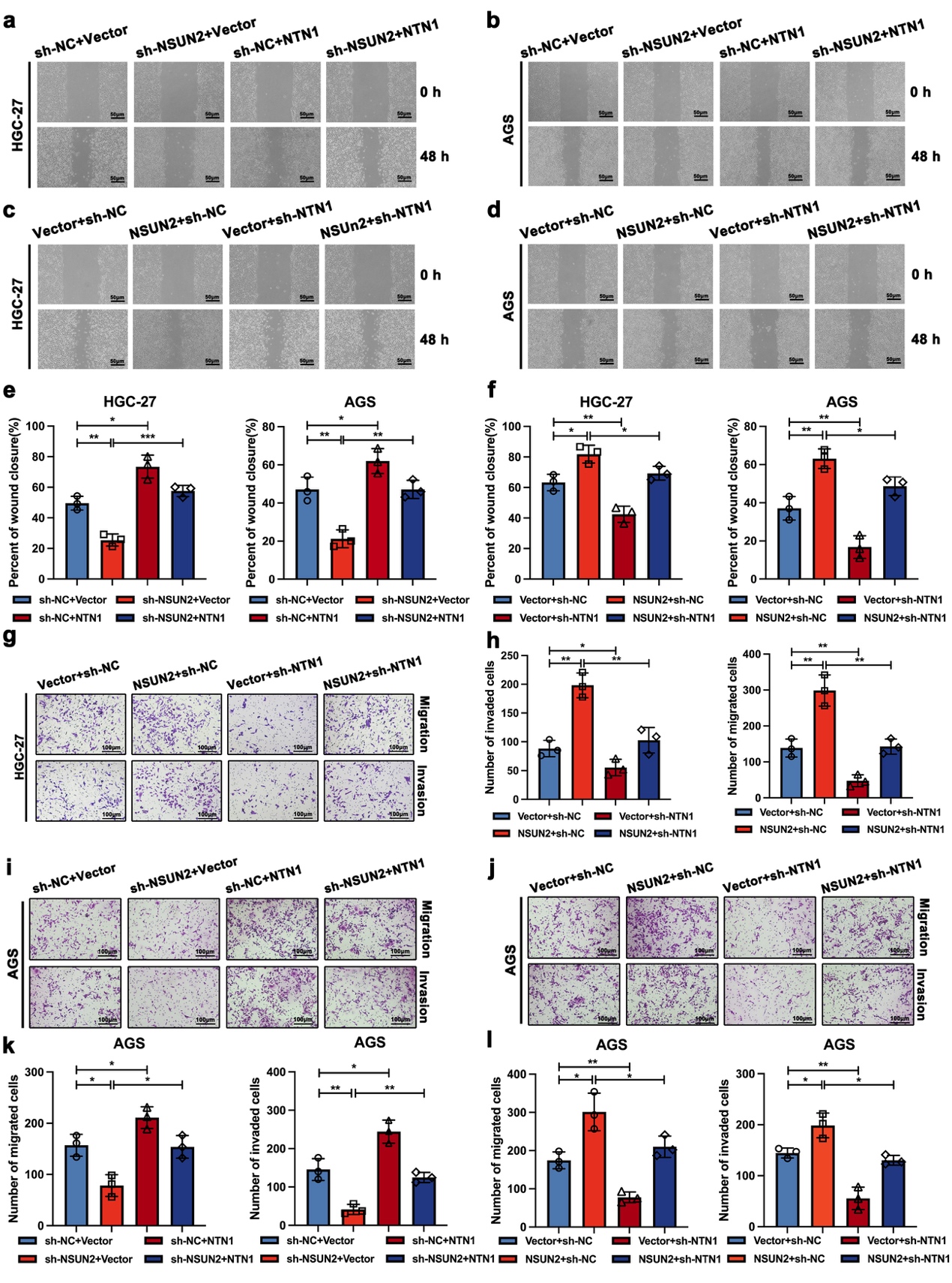


**a-f** Indicated HGC-27 and AGS cells were used to perform wound healing assay and corresponding statistical graphs were shown. Scale bars: 50μm. **g-l** Transwell assay including migration and invasion were performed utilizing indicated HGC-27 and AGS cells. Corresponding statistical graphs were shown. Scale bars: 100μm. Data and error bars were shown as mean ± SD of triplicate independent replicate experiments and all data were analyzed by Student's t test. (*P < 0.05; **P < 0.01; ***P < 0.001).

**Supplementary Figure. S8**


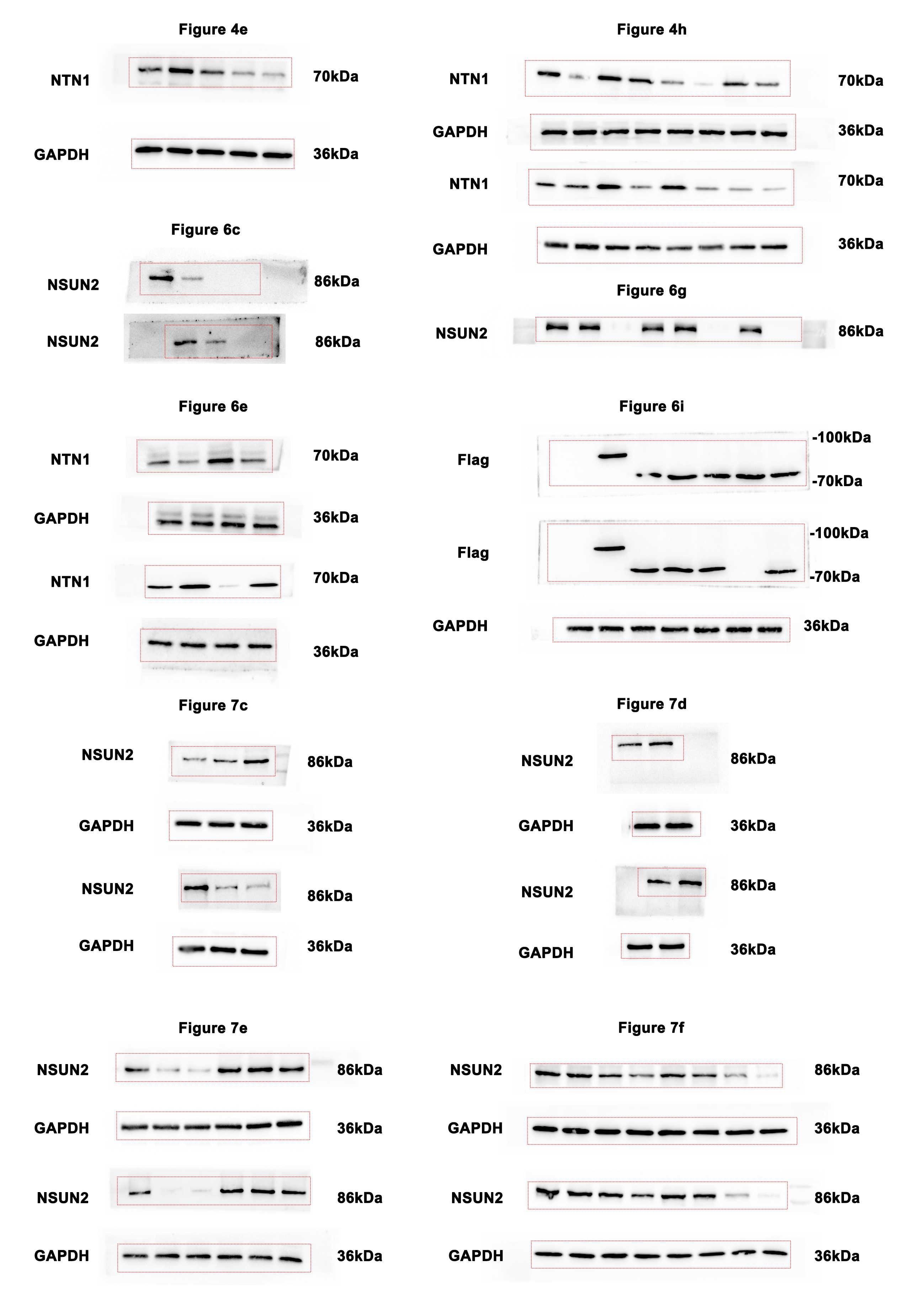


The uncropped scans of western blots and gels from the figures of the main manuscript.

**Supplementary Figure. S9**


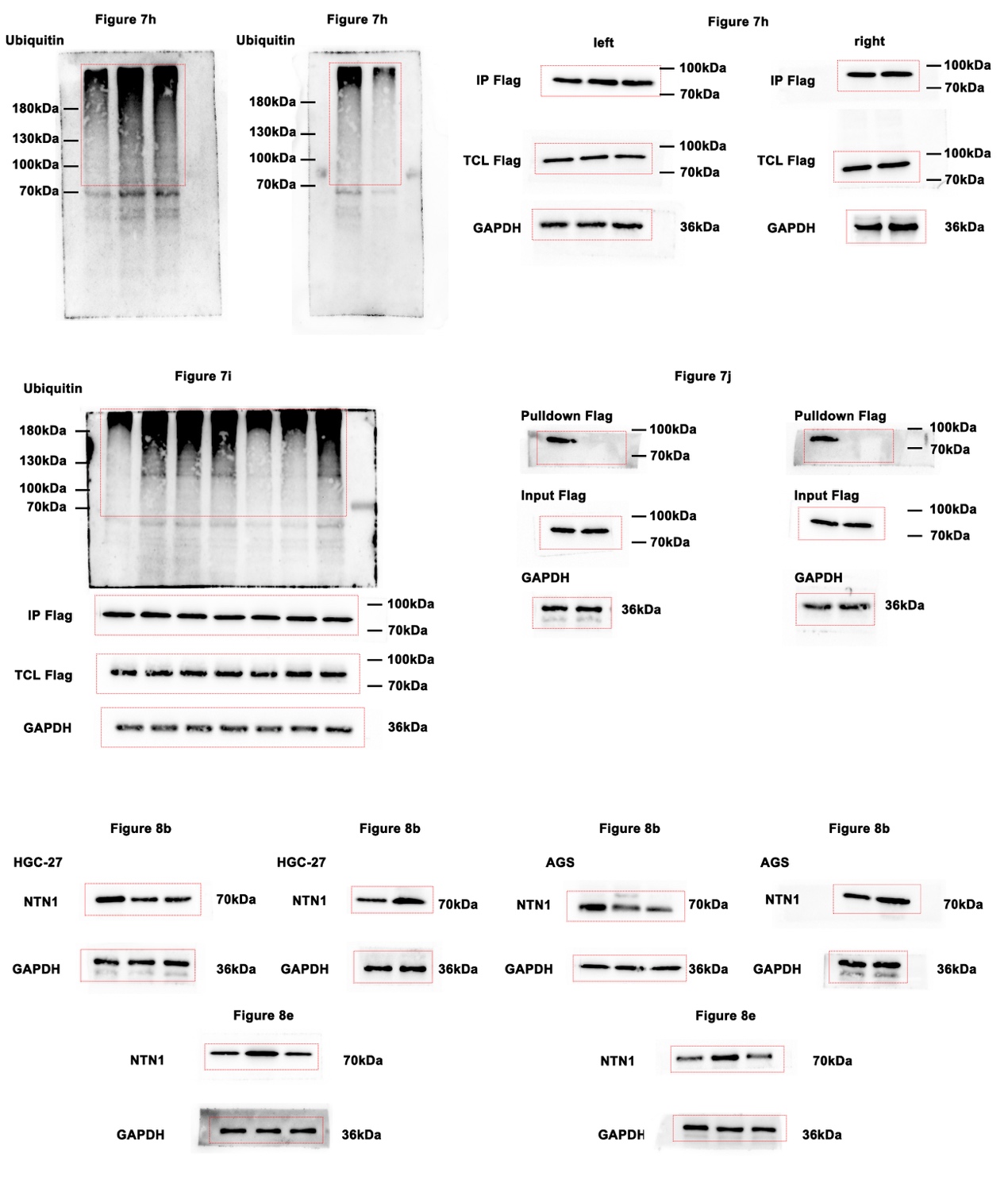


The uncropped scans of western blots and gels from the figures of the main manuscript.

**Supplementary Figure. S10**


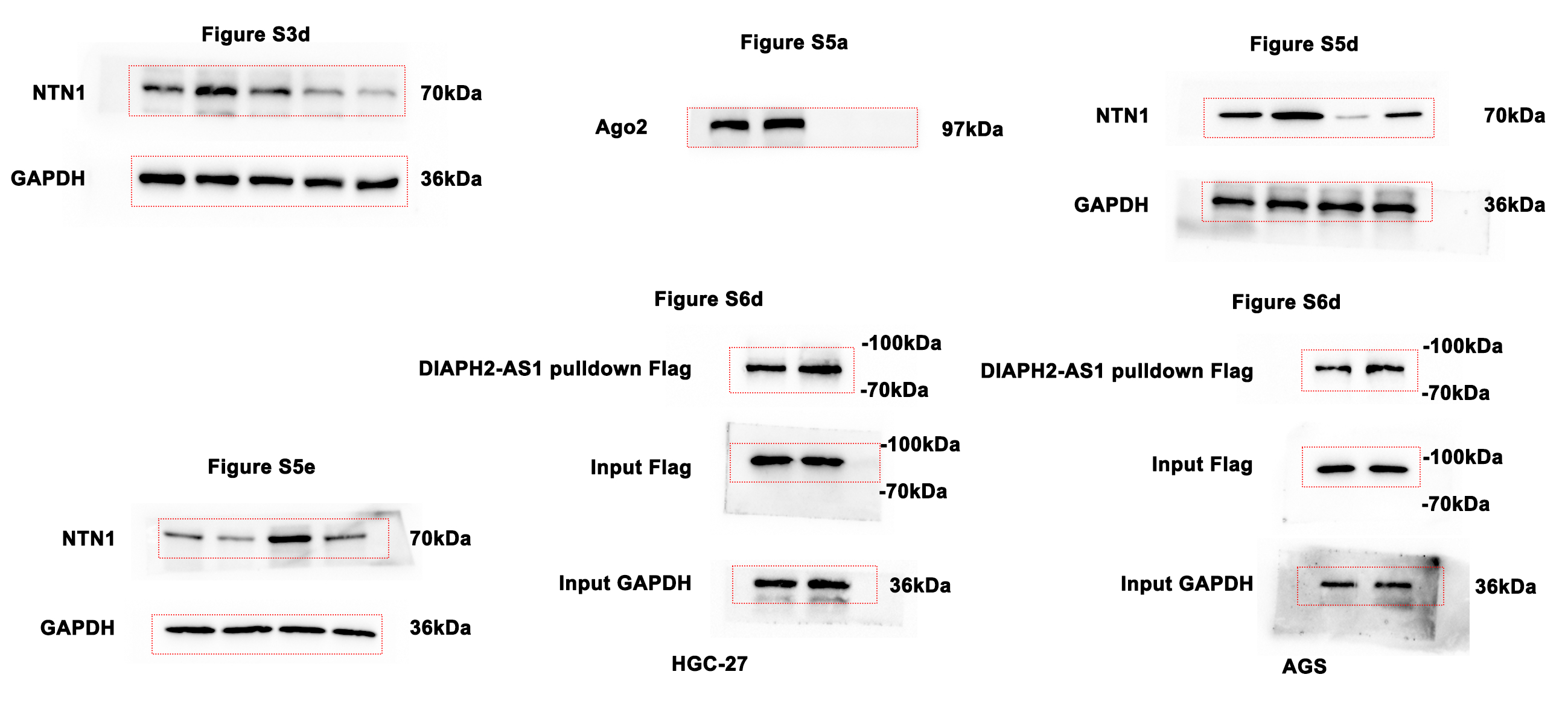


The uncropped scans of western blots and gels from the supplementary figures.

**Supplementary Table S1**

**Primers used in this study**

| NAALADL2-AS1 | Forward | TGAATGAGCGACACAGGATGA |
| --- | --- | --- |
|  | Reverse | CCCAACTGTGGTTGGTGATG |
| LOC101928030 | Forward | GAACCGGGAGCTGAATGTGA |
|  | Reverse | TCCTGGCAGATTGGGCTCTA |
| LOC101927082 | Forward | GAAAGCCTGGACTTTTGAGCC |
|  | Reverse | TTCCCTGATATGTGGGCAGG |
| LINC01831 | Forward | TGGAAGGCAAGCATACGGAA |
|  | Reverse | TGGAGAGGCATTTCCACACG |
| LINC01392 | Forward | ACACTGTGCCAGGTTCCAAAT |
|  | Reverse | AGTGGAAGCTTCGGATTTCCC |
| DIAPH2-AS1 | Forward | TCTCTGACTCAACCTTTGACACT |
|  | Reverse | TGGACTATATCTTGGAAGACGGA |
| LOC105370954 | Forward | CAGCGGATTTTCTCCCCGAT |
|  | Reverse | CAGGCAAGATCCAGTCTGCT |
| TAB3-AS1 | Forward | TTTTCTAATGAATTCTGAGCCCAC |
|  | Reverse | GCACCATGCTTTGGATTCTGG |
| LOC101929148 | Forward | AGACCAGATCGAGGCAGACT |
|  | Reverse | GCCTCATGGGACTATCACGG |
| LOC102723883 | Forward | TGGGTGGACGTTCTTTCCTG |
|  | Reverse | TGCTTTTCCTCGCTCCAAGT |
| PABPC5-AS1 | Forward | CCCAGATACCCAAGATGCCC |
|  | Reverse | AGCTCCCGAGGGCCTATATT |
| LOC105377962 | Forward | GCCTGCAATCCACTGGAAAC |
|  | Reverse | TCCCCAACTTGTACTGCCTC |
| NTN1 | Forward | CTATGTGGGAGGGAGGGACA |
|  | Reverse | CATGGCCCACAGGAATGTCT |
| NSUN2 | Forward | GCTACCCCGAGATCGTCAAG |
|  | Reverse | CTTTCTGACCGTCCACCTCC |
| U6 | Forward | CTCGCTTCGGCAGCACA |
|  | Reverse | AACGCTTCACGAATTTGCGT |
| GAPDH | Forward | CAATGACCCCTTCATTGACC |
|  | Reverse | TTGATTTTGGAGGGATCTCG |

**Supplymentary Table S2**

**Antibodies used in this study**

| **Antibody** | **Company (Art.No.)** | **Apply (dilution)** |
| --- | --- | --- |
| PGP 9.5 | Abcam (ab108986) | IF (1:500)  IHC (1:500) |
| NTN1 | Proteintech (20235-1-AP) | WB (1:1000) |
| NSUN2 | Proteintech (20854-1-AP) | WB (1:1000)  IP (0.5-4.0 ug) |
| Argonaute-2 | Abcam (ab186733) | WB (1:1000) |
| Anti-FLAG | Sigma-Aldrich (F1840) | WB (1:1000) |
| Anti-HA | Proteintech (51064-2-AP) | WB (1:1000) |
| GAPDH | Abcam (ab8245) | WB (1:2000) |

**Supplementary Table S3**

**siRNAs used in this study**

| **Genes** | **Sequence** |
| --- | --- |
| si-DIAPH2-AS1#1 | AUAAACAUUCGAACUAUGGAA |
| si-DIAPH2-AS1#2 | UUCUUAGUCUCCUUUAAUGUG |
| si-DIAPH2-AS1#3 | AAAAAUAUAUAUAUAUAUGCU |
